# Neutrophil-specific expression of JAK2-V617F or CALRmut induces distinct inflammatory profiles in myeloproliferative neoplasia

**DOI:** 10.1186/s13045-024-01562-5

**Published:** 2024-06-09

**Authors:** Tobias Ronny Haage, Emmanouil Charakopoulos, Vikas Bhuria, Conny K. Baldauf, Mark Korthals, Juliane Handschuh, Peter Müller, Juan Li, Kunjan Harit, Gopala Nishanth, Stephanie Frey, Martin Böttcher, Klaus-Dieter Fischer, Jan Dudeck, Anne Dudeck, Daniel B. Lipka, Burkhart Schraven, Anthony R. Green, Andreas J. Müller, Dimitrios Mougiakakos, Thomas Fischer

**Affiliations:** 1 Department of Hematology, Oncology, and Cell Therapy, Medical Faculty, Otto-von-Guericke University, Leipziger Str. 44, 39120 Magdeburg, Germany; 2 Healthcampus Immunology, Inflammation and Infectiology (GC-I, Otto-von-Guericke-University, Magdeburg, Germany; 3 Institute for Molecular and Clinical Immunology, Medical Faculty, Otto-von-Guericke University, Magdeburg, Germany; 4 Institute for Biochemistry and Cell Biology, Medical Faculty, Otto-von-Guericke University, Magdeburg, Germany; 5 Center for Health and Medical Prevention – CHaMP, Otto-von-Guericke University, Magdeburg, Germany; 6 Cambridge Stem Cell Institute, Department of Haematology, University of Cambridge, Cambridge, GB England; 7 Institute for Medical Microbiology and Hospital Epidemiology, Hannover Medical School, Hannover, Germany; 8 Section of Translational Cancer Epigenomics, Division of Translational Medical Oncology, German Cancer Research Center (DKFZ), National Center for Tumor Diseases (NCT) Heidelberg, Heidelberg, Germany; 9 Faculty of Medicine, Otto-von-Guericke University, Magdeburg, Germany; 10 Helmholtz Centre for Infection Research, Braunschweig, Germany

**Keywords:** MPN, Inflammation, Neutrophils, JAK2-V617F, CALR mutations

## Abstract

**Background:**

Neutrophils play a crucial role in inflammation and in the increased thrombotic risk in myeloproliferative neoplasms (MPNs). We have investigated how neutrophil-specific expression of JAK2-V617F or CALRdel re-programs the functions of neutrophils.

**Methods:**

Ly6G-Cre JAK2-V617F and Ly6G-Cre CALRdel mice were generated. MPN parameters as blood counts, splenomegaly and bone marrow histology were compared to wild-type mice. Megakaryocyte differentiation was investigated using lineage-negative bone marrow cells upon in vitro incubation with TPO/IL-1β. Cytokine concentrations in serum of mice were determined by Mouse Cytokine Array. IL-1α expression in various hematopoietic cell populations was determined by intracellular FACS analysis. RNA-seq to analyse gene expression of inflammatory cytokines was performed in isolated neutrophils from JAK2-V617F and CALR-mutated mice and patients. Bioenergetics of neutrophils were recorded on a Seahorse extracellular flux analyzer. Cell motility of neutrophils was monitored in vitro (time lapse microscopy), and in vivo (two-photon microscopy) upon creating an inflammatory environment. Cell adhesion to integrins, E-selectin and P-selection was investigated in-vitro. Statistical analysis was carried out using GraphPad Prism. Data are shown as mean ± SEM. Unpaired, two-tailed t-tests were applied.

**Results:**

Strikingly, neutrophil-specific expression of JAK2-V617F, but not CALRdel, was sufficient to induce pro-inflammatory cytokines including IL-1 in serum of mice. RNA-seq analysis in neutrophils from JAK2-V617F mice and patients revealed a distinct inflammatory chemokine signature which was not expressed in CALR-mutant neutrophils. In addition, IL-1 response genes were significantly enriched in neutrophils of JAK2-V617F patients as compared to CALR-mutant patients. Thus, JAK2-V617F positive neutrophils, but not CALR-mutant neutrophils, are pathogenic drivers of inflammation in MPN. In line with this, expression of JAK2-V617F or CALRdel elicited a significant difference in the metabolic phenotype of neutrophils, suggesting a stronger inflammatory activity of JAK2-V617F cells. Furthermore, JAK2-V617F, but not CALRdel, induced a VLA4 integrin-mediated adhesive phenotype in neutrophils. This resulted in reduced neutrophil migration in vitro and in an inflamed vessel. This mechanism may contribute to the increased thrombotic risk of JAK2-V617F patients compared to CALR-mutant individuals.

**Conclusions:**

Taken together, our findings highlight genotype-specific differences in MPN-neutrophils that have implications for the differential pathophysiology of JAK2-V617F versus CALR-mutant disease.

**Supplementary Information:**

The online version contains supplementary material available at 10.1186/s13045-024-01562-5.

## Background

The discovery of JAK2-V617F, an activating somatic gain-of-function mutation of the Janus kinase 2 (JAK2), in classic Philadelphia chromosome-negative chronic myeloproliferative neoplasms (MPNs) such as essential thrombocythemia (ET), polycythemia vera (PV), and primary myelofibrosis (PMF), has provided first insight into the molecular basis of MPN. Genomic analysis revealed the occurrence of JAK2-V617F in the majority (95%) of PV patients and in 50% of ET and PMF patients [1–3]. Somatic mutations within the Calreticulin (*CALR*) gene, a major endoplasmic reticulum (ER) chaperone, are detected in 60–80% of JAK2 and thrombopoietin receptor (MPL) non-mutated ET and PMF patients [4, 5]. Calreticulin mutations are located in exon 9 and are classified as either type 1 mutation, exemplified by a 52 bp deletion, or type 2 mutation, exemplified by a 5 bp insertion [5]. All CALR mutations generate a novel C-terminus in the mutant protein [6]. Notably, in hematopoietic cells, CALR mutations (CALRmut) have been associated with an indirect activation of the JAK2-STAT pathway via MPL, accomplished through binding of mutated CALR to its extracellular domain [7–9]. However, due to CALR’s chaperone functions in the ER, CALRmut also activates additional signaling pathways. These include the activation of the unfolded protein response [10–12], defective interaction of CALRmut with the store-operated calcium entry (SOCE) machinery [13] and modulation of binding to known and as yet unidentified partners [14]. JAK2-V617F and CALRmut are the most frequent genetic alterations in MPNs and are responsible for driving the pathophysiology of the disease [1, 5, 15]. The genetic signature predicts clinical phenotypes such as abnormal blood counts, risk of leukemic transformation and event-free survival [16].

A key characteristic of JAK2- and CALR-mutated MPN is a chronic non-resolving inflammatory condition that promotes clonal evolution [17–20]. Importantly, recent research has revealed that chronic inflammation in MPN is indeed a driver of leukemic transformation [21]. In addition, a major cause of morbidity and mortality in MPN is venous and arterial thrombosis. In PV, large clinical studies revealed an incidence of 20–23% thrombotic events at diagnosis [22–24]. However, according to a recent meta-analysis in PMF, CALR-mutated patients demonstrated a lower risk of thrombosis than JAK2-mutated patients [25]. In myelofibrosis, CALR-mutated patients also displayed a distinct disease phenotype compared to JAK2-V617 positive individuals: CALR mutated patients presented with less frequent inflammatory symptoms upon diagnosis, fewer thrombotic complications and less prominent splenomegaly [26]. A similar genotype-phenotype correlation is evident in ET: CALR mutated individuals are substantially different from JAK2-V617F positive patients in terms of hematologic and clinical features [27]. Furthermore, despite having lower platelet (PLT) counts, JAK2-V617F positive ET patients have twice the risk of thrombotic complications compared to those with CALR mutations [27]. This paradoxical observation suggests that JAK2-V617F induced alterations in adhesion properties of granulocytes, erythrocytes, PLTs and endothelial cells might be more relevant to the pathogenesis of thrombosis than the PLT counts per se [28–30]. This view is supported by research on clinical samples, which revealed high neutrophil-PLT aggregates in ET and PV patients, as well as increased activation of PLTs, leukocytes, and coagulation in MPNs and increased adhesion of JAK2-V617F positive granulocytes [31–33]. These findings suggest significant differences in the regulation of inflammation and pro-thrombotic risk between JAK2-V617F and CALR-mutated MPN patients.

Neutrophils are the most abundant leukocytes in the organism and therefore an important source of inflammatory cytokines. They are increasingly recognized as an important pathogenic link to chronic non-resolving inflammation and the pro-thrombotic risk in MPN [34–37]. MPN neutrophils display a malignant phenotype and contribute to a number of major pathological incidents in MPNs [34, 38]. Importantly, previous research has shown that JAK2-V617F positive neutrophils exhibit a pro-adhesive phenotype, which contributes to the marked pro-thrombotic state of JAK2-V617F positive MPN [39]. However, there is almost no data comparing the activities of CALR-mutated neutrophils versus JAK2-V617F positive neutrophils side-by-side in MPNs. Here, we investigated a range of inflammatory conditions induced by neutrophil-specific expression of JAK2-V617F or CALRmut, which we hypothesized were related to patients’ inflammatory symptoms and pro-thrombotic risks.

## Materials and methods

### Sex as a biological variable

Our study examined male and female animals, and similar findings are reported for both sexes.

### Mouse models

The Ly6G-Cre JAK2-V617F mouse model was generated by crossing floxed heterozygous *JAK2*^*+/loxP−VF−loxP*^ mice with *Ly6G*^*+/Cre−tdTomato*^*Catchup*^*IVM−red*^ mice. *JAK2*^*+/loxP−VF−loxP*^ mice were kindly provided by Ann Mullally (Harvard Medical School, Boston, Massachusetts) [40]. Experiments were conducted using *JAK2*^*+/loxP−VF−loxP*^*Ly6G*^*+/Cre−tdTomato*^*Catchup*^*IVM−red*^ (Ly6G-Cre *JAK2*^*+/VF*^) and *JAK2*^*+/+*^*Ly6G*^*+/Cre−tdTomato*^*Catchup*^*IVM−red*^ (Ly6G-Cre *JAK2*^*+/+*^) mice as wild type (WT) controls [41]. Exon 1 of the *Ly6g*-gene was replaced by a knock-in allele of Cre recombinase and tdTomato leading to neutrophil-specific expression of JAK2-V617F. An additional Cre recombinase-depending expression of tdTomato was achieved through a CAG promoter located in the *ROSA26* locus [41].

The Ly6G-Cre CALRdel mouse model was generated by crossing heterozygous mice harboring the loxP flanked CALRdel52 mouse-human chimeric oncogene in exon 9 (*CALR*^*+/loxP−exon9−loxP*^) with Ly6G-Cre mice [42]. Experiments were conducted using *CALR*^*+/loxP−exon9−loxP*^*Ly6g*^*+/Cre−tdTomato*^*Catchup*^*IVM−red*^ (Ly6G-Cre *CALR*^*+/del*^) and *CALR*^*+/+*^*Ly6g*^*+/Cre−tdTomato*^*Catchup*^*IVM−red*^ (Ly6G-Cre CALR^+/+^) mice as WT controls. Neutrophil-specific expression of CALRdel52 was induced by replacement of exon 1 of the *Ly6g*-gene by a knock-in allele of Cre recombinase and tdTomato. All mice used exhibited a CAG promoter-driven additional expression of tdTomato from the *ROSA26* locus in a Cre-dependent manner.

The Vav-Cre JAK2-V617F and CALRdel model have been described previously [42, 43].

### Blood count analysis

Blood count analysis was obtained by an automatic blood counting machine (ADVIA 2120 systems, Siemens, Germany).

### Flow cytometry analysis

Immunophenotyping by flow cytometry was performed using a FACSCanto II (BD Biosciences). Primary antibodies used are listed in Additional File 1: Table S1. Due to overlapping fluorescent spectra of tdTomato and phycoerythrin (PE), PE-conjugated antibodies were not used in Ly6G-Cre mice.

### Bone marrow and spleen sections

Hematoxylin-eosin staining was performed on bone marrow (BM) and spleen sections. After deparaffinization and rehydration, sections were stained with hematoxylin and eosin.

### Analysis of hematopoietic stem and progenitor cells (HSPCs)

Isolating HSPCs and quantitative analysis was performed as previously described [43, 44].

### Measurement of inflammatory cytokines

Cytokine concentrations in serum were determined by Eve Technologies, Canada (Mouse Cytokine Array/Chemokine Array 32-Plex, duplicate testing) using two-fold diluted serum samples.

### Metabolic flux analyses

Bioenergetics of neutrophils were recorded on a Seahorse XFe96 extracellular flux analyzer (Agilent Technologies, St. Clara, CA) as described previously [45, 46]. Details of the method are outlined in Additional file 2: Supplemental Methods.

### In vitro time lapse recording of cell motility

BM cells were harvested from femur and tibia and neutrophils were isolated by negative selection [47] followed by fluorescence-activated cell sorting (FACS) using their tdTomato autofluorescence. Four wells of a 15-well angiogenesis µ-slide (Ibidi) were precoated overnight with ICAM-1 and VCAM-1 (5 µg/mL, R&D Systems). Overall, 50,000 tdTomato expressing neutrophils were resuspended and seeded per well in 1xHBSS (Biochrom) containing 0.2% fatty acid-free BSA (Roth) and 1 mM HEPES (Life Technologies). The slide was subsequently transferred to the microscope stage in a 5% CO_2_ atmosphere at 37 °C. Immediately after settling of the cells, time lapse recording was started using a Leica DMI6000 widefield microscope (40x objective; LAS AF software, version 2.0.2, Leica Microsystems, Germany). Brightfield and tdTomato fluorescence images were captured every 37 s in four wells at a time using automated XY-stage positioning over a period of one hour. Manual tracking of 50 cells per mouse was done with ImageJ (version v1.52n) and the ImageJ plugin MTrackJ (version 1.5.1).

### Saphenous vein stenosis model

Partial ligation of the great saphenous vein (GSV) [48] was performed on 10 to 16 weeks old male Ly6G-Cre *JAK2*^*+/VF*^ and *JAK2*^*+/+*^ mice. Anesthetized mice (Ketamin 100 mg/kg bodyweight and Xylazine/Rompun 10 mg/kg bodyweight) were fixated on the back on a 37 °C temperature-controlled heating pad. After removing the fur of the inner thigh, the GSV was microsurgically exposed under microscopic view (Leica S8 APO) and partially ligated using a 7 − 0 polypropylene suture. To avoid complete venous occlusion, a part of a needle tip (30 gauge; equivalent to a diameter of 0.255 mm) was inserted as placeholder and immediately removed after applying the partial ligation.

### In vivo two-photon (2P) microscopy

In vivo two-photon (2P) microscopy was performed as previously described [49, 50] and is outlined in Additional file 2: Supplemental Methods.

### Statistics

Statistical analysis was carried out using GraphPad Prism (version 9). Data are shown as mean ± SEM. Unpaired, two-tailed t-tests were applied. For comparison of gene expression between the *JAK2*^*+/+*^ versus *JAK2*^*+/VF*^ and *CALR*^*+/+*^ versus *CALR*^*+/del*^ groups of samples, the DESeq2 software was used.

Gene set enrichment analysis was performed using the GSEAv4.3.2 (UC San Diego and Broad Institute; https://www.gsea-msigdb.org). Comparisons exhibiting a p-value < 0.05 and FDR q-value < 0.15 were considered significant.

## Results

### Neutrophil-specific expression of *JAK2-V617F*, but not *CALRdel*, induces thrombocytosis and megakaryocyte hyperplasia

**Fig. 1:**
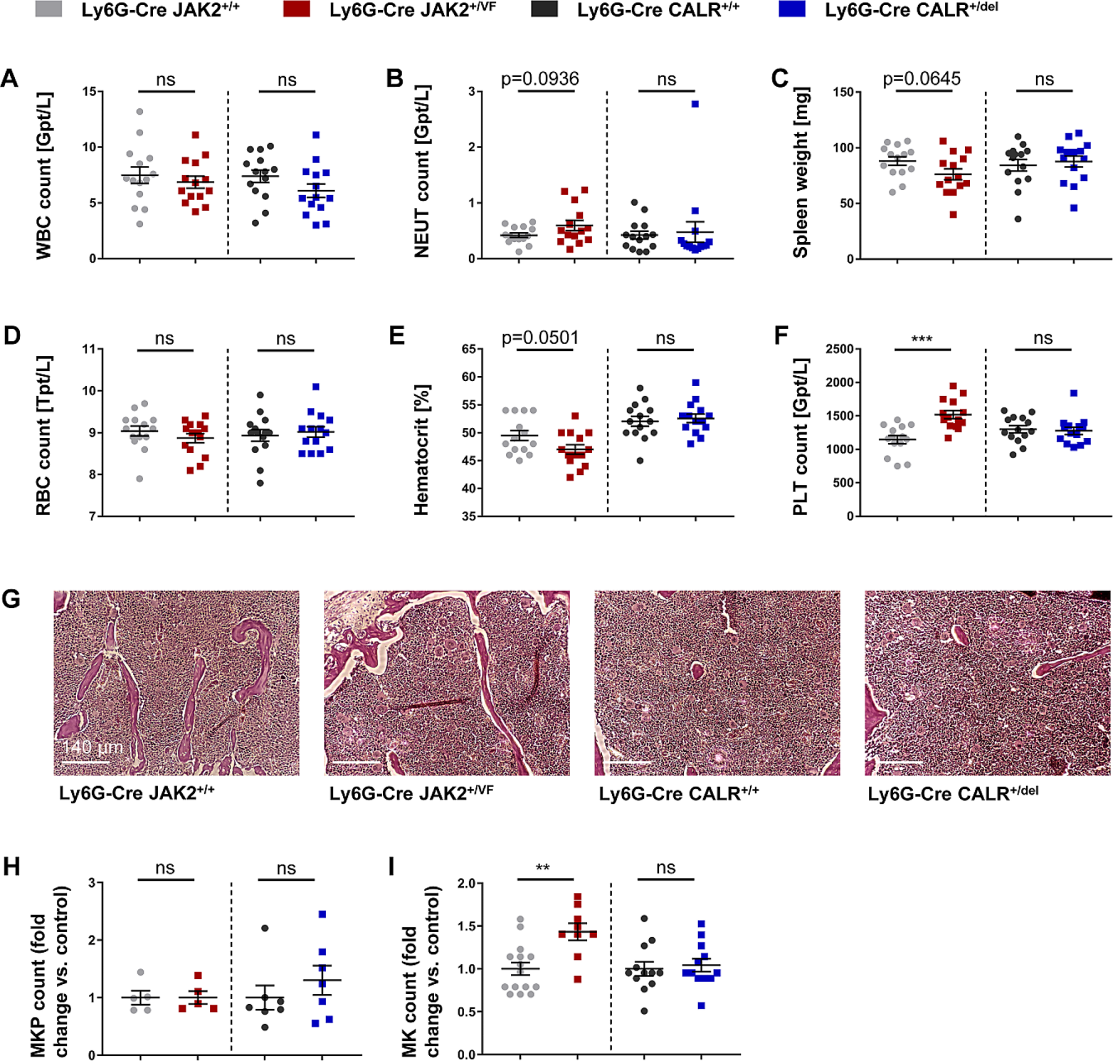
Neutrophil-specific expression of *JAK2-V617F*, but not *CALRdel*, induces thrombocytosis and megakaryocyte hyperplasia. (**A-F**) White blood cell (WBC) count, neutrophil (NEUT) count, spleen weight, red blood cell (RBC) count, hematocrit, and platelet (PLT) count of Ly6G-Cre *JAK2*^*+/+*^, *JAK2*^*+/VF*^, *CALR*^*+/+*^ and *CALR*^*+/del*^ mice (each *n* = 14). (**G**) Representative hematoxylin-eosin staining of bone marrow sections of *JAK2*^*+/+*^ (*n* = 5), *JAK2*^*+/VF*^ (*n* = 3), *CALR*^*+/+*^ (*n* = 8) and *CALR*^*+/del*^ mice (*n* = 10). (**H**) Megakaryocyte progenitor (MKP) counts in bone marrow of Ly6G-Cre *JAK2*^*+/+*^ (*n* = 5), *JAK2*^*+/VF*^ (*n* = 5), *CALR*^*+/+*^ (*n* = 7) and *CALR*^*+/del*^ mice (*n* = 7) shown as fold change versus control. (**I**) Quantitative analysis (performed in a blinded fashion) of megakaryocytes (MKs) in bone marrow of Ly6G-Cre *JAK2*^*+/+*^, *JAK2*^*+/VF*^, *CALR*^*+/+*^ and *CALR*^*+/del*^ mice in 200x high-power fields (HPF) showing numbers of MKs/HPF as fold change versus control. Data are shown as mean ± SEM. **p ≤ 0.01, ***p ≤ 0.001 (unpaired, two-tailed t-test)

Aside from their crucial role in innate immunity, neutrophils have an important and previously underestimated function in the regulation of hematopoietic stem cell and erythropoietic niches in both normal physiologic conditions and MPN pathophysiology [51, 52]. To examine the effects of neutrophil-restricted expression of JAK2-V617F and of CALRdel in inflammation, Ly6G-Cre positive *JAK2*^*+/VF*^, *JAK2*^*+/+*^, *CALR*^*+/del*^ and *CALR*^*+/+*^ mice were generated as described under Material and Methods. Cre recombinase-depending expression of tdTomato and of the transgene was achieved through a CAG promoter located in the *ROSA26* locus [41]. Control experiments demonstrated that JAK2-V617F or CALRdel is only expressed in tdTomato positive neutrophils of Ly6G-Cre *JAK2*^*+/VF*^ and *CALR*^*+/del*^ mice, respectively (Additional file 3: Fig. S1). The neutrophil-specificity of the Ly6G-Cre model (Catchup model) has been described previously [41]. Importantly, Ly6G-Cre directed tdTomato expression was absent in CD11b + Ly6G^−^ macrophages or other Ly6G^−^ leukocytes and in GMPs [41]. Additional control experiments in hematopoietic progenitors, in monocytes, in the erythrocyte lineage, platelets and in megakaryocyte progenitors (MKPs) demonstrated almost complete tdTomato-negativity in Ly6G-Cre *JAK2*^*+/VF*^ and Ly6G-Cre *CALR*^*+/del*^ mice, respectively (Additional file 3: Fig. S2-4). In Ly6G-Cre *JAK2*^*+/VF*^ and CALRdel (*CALR*^*+/del*^) mice, white blood cell (WBC) counts and red blood cell (RBC) counts remained within the normal range (Fig. 1, A and D). However, there was a tendency towards an increase in neutrophil counts in *JAK2*^*+/VF*^ mice, while hematocrit (HCT) and spleen weight tended to decrease in these mice (Fig. 1, B, C and E). Interestingly, there was a moderate but highly significant increase in PLT counts of *JAK2*^*+/VF*^ mice (*JAK2*^*+/VF*^: 1519 ± 59 10^9^/L; *JAK2*^*+/+*^: 1146 ± 58 10^9^/L) in both male and female mice (Fig. 1F and Additional file 3: Fig. S5, A-C). Increased PLT counts were also observed in aged *JAK2*^*+/VF*^ mice (Additional file 3: Fig. S5F). BM sections of *JAK2*^*+/VF*^ mice exhibited megakaryocyte (MK) hyperplasia and formation of MK clusters, which are regarded as a hallmark of MPN [40, 53] (Fig. 1, G and I). Nonetheless, MK progenitor counts were comparable (Fig. 1H). Serum thrombopoietin (TPO) concentrations showed no discrepancies between the two mouse strains (Additional file 3: Fig. S5G). MK hyperplasia in *JAK2*^*+/VF*^ mice was not caused by an increase in megakaryocyte/erythroid progenitors (MEP) in the BM (Additional file 3: Fig. S6A and C). Phenotypic analysis of HSPCs revealed that expression of JAK2-V617F or CALRdel in neutrophils did not result in any significant numerical or compositional changes of HSPCs in the BM or the splenic compartment (Additional file 3: Fig. S6, A-D). Additionally, examination of spleens did not reveal any discernible differences in spleen size or composition between both Ly6G-Cre *JAK2*^*+/VF*^ and *CALR*^*+/del*^ mice and their respective WT controls (Additional file 3: Fig. S7).

### Neutrophil-specific expression of *JAK2-V617F*, but not *CALRdel*, up-regulates inflammatory cytokines in serum

**Fig. 2:**
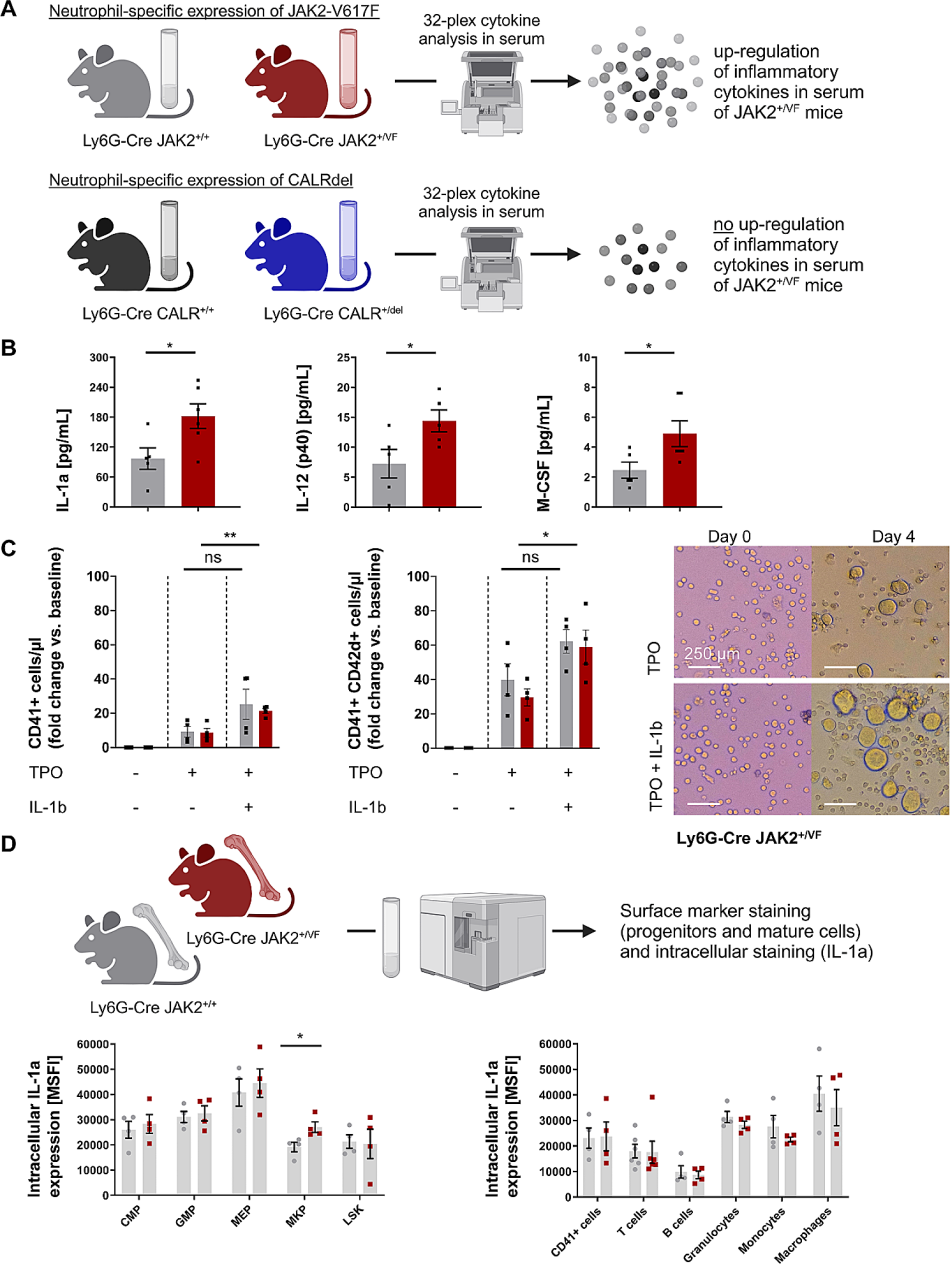
Neutrophil-specific expression of *JAK2-V617F*, but not *CALRdel*, up-regulates inflammatory cytokines. (**A**) Cartoon depicting the experimental design to study serum cytokine concentrations in *JAK2*^*+/VF*^ and *CALR*^*+/del*^ mice in comparison to their corresponding WT controls. Cytokine protein concentrations in serum of Ly6G-Cre *JAK2*^*+/+*^ (*n* = 6) and *JAK2*^*+/VF*^ mice (*n* = 6), and in serum of Ly6G-Cre *CALR*^*+/+*^ (*n* = 8) and *CALR*^*+/del*^ mice (*n* = 10) were analyzed by Eve Technologies, Canada (Mouse Cytokine Array/Chemokine Array 32-Plex, duplicate testing). Created with Biorender.com. (**B**) Bar graphs of significantly elevated serum cytokine concentrations of IL-1α, IL-12(p40) and M-CSF in Ly6G-Cre *JAK2*^*+/VF*^ mice. Data are shown as median ± IQR. *p ≤ 0.05 (unpaired, two-tailed t-test). (**C**) IL-1β induced megakaryocytic differentiation of lineage-negative cells isolated from bone marrow. Left and middle panel: Impact of IL-1β (25 ng/ml) on the number of formed immature (CD41^+^) and mature (CD41^+^ CD42d^+^) megakaryocytes upon TPO-driven differentiation of lineage-negative cells isolated from bone marrow of Ly6G-Cre *JAK2*^*+/+*^ and *JAK2*^*+/VF*^ mice (each *n* = 4). Data are shown as mean ± SEM. *p *≤* 0.05 (unpaired, two-tailed t-test). Right panel: Representative images of megakaryocytes differentiated from lineage-negative bone marrow cells isolated from Ly6G-Cre *JAK2*^*+/+*^ and *JAK2*^*+/VF*^ mice at baseline and upon four-day TPO-driven differentiation with or without IL-1β (*n* = 2). (**D**) Neutrophil-specific expression of *JAK2-V617F* increases IL-1α expression in megakaryocyte progenitors (MKP). Intracellular staining for IL-1α levels in various hematopoietic cell populations using anti-IL-1α antibody and isotype control antibody, respectively was performed as described under Supplemental Methods. Mean fluorescence intensity (MFI) was measured by flow cytometry. The specific MFI (MSFI) was calculated by subtracting the MFI of the isotype control from the MFI of the anti-IL-1α antibody stained sample. Data are shown as mean ± SEM. *p ≤ 0.05 (unpaired, two-tailed t-test with Welch correction). Cartoon created with Biorender.com

A chronic non-resolving inflammatory syndrome with a pro-inflammatory cytokine signature in the serum is a significant disease feature in MPNs [54, 55]. However, the role of granulocytes in the inflammatory condition is not well-understood. To investigate whether expression of JAK2-V617F or CALRdel specifically in neutrophils induces pro-inflammatory cytokines, we measured serum concentrations of a panel of cytokines in Ly6G-Cre *JAK2*^*+/VF*^ and *CALR*^*+/del*^ mice (Fig. 2A). Compared to the WT controls, the cytokine levels of IL-1α, IL-12 (p40), and M-CSF cytokines were significantly (*p* < 0.05) elevated in *JAK2*^*+/VF*^ mice by factors of 1.8, 1.6 and 2.1, respectively (Fig. 2B and Additional file 1: Tab. S2). Furthermore, in Ly6G-Cre *JAK2*^*+/VF*^ mice, although not statistically significant, levels of five additional cytokines in serum were observed to be upregulated by more than 1.5-fold: IL-1β, IL-2, IL-10, IL-17, and TNFα (Additional file 1: Tab. S2). In contrast to the JAK2-V617F mutation, statistically significant changes in the serum cytokine levels were not induced by neutrophil-specific expression of CALRdel and levels of only two cytokines were up-regulated by more than 1.5-fold: IL-5 and LIX (CXCL5) (Additional file 1: Tab. S3). IL-5 has a significant role in type-2 adaptive immunity, which includes atopic diseases, but has not yet been implicated in cancer-associated inflammation [56]. Together, this suggests that expression of JAK2-V617F in neutrophils is sufficient to induce typical MPN pro-inflammatory cytokines in serum [17–20]. Conversely, expression of CALRdel in neutrophils seems to have no significant impact on the induction of a pro-inflammatory cytokine signature in serum.

Recent studies revealed that IL-1β has a pivotal role in clonal expansion, BM fibrosis and amplified megakaryopoiesis associated with JAK2-V617F-driven MPN-like disease in mice [17, 18]. Both, the genetic deletion of IL-1β and of IL-1R1, respectively and administration of anti-IL-1β antibody and anti-IL-1R1 antibody, respectively suppressed increased PLT and BM CD41^+^ cell counts in JAK2-V617F driven MPN-mice [17, 18]. Therefore, we hypothesized that the higher PLT counts observed in the Ly6G-Cre JAK2-V617F model were due to the increased IL-1β serum levels (Additional file 1: Tab. S2). Thus, we studied the impact of IL-1β on the differentiation of lineage-negative (lin^−^) BM cells into MKs. Figure 2C displays that IL-1β increased TPO-driven megakaryopoiesis in vitro using lin^−^ BM cells isolated from Ly6G-Cre *JAK2*^*+/+*^ and *JAK2*^*+/VF*^ mice. Ploidy analysis additionally indicated an increase in 8 N-cells following IL-1β stimulation (Supplementary Figure S8). Considering the published evidence [17, 18] regarding the significant role of IL-1β in JAK2-V617F-induced thrombocytosis and elevated megakaryopoiesis, our findings support the concept that increased IL-1β serum levels are responsible for the observed elevation of PLT and MK counts in Ly6G-Cre *JAK2*^*+/VF*^ mice.

Next, we investigated which hematopoietic cell population is the source of the elevated serum levels of inflammatory cytokines in JAK2-V617F induced MPN-like disease. We focused on IL-1α and performed intracellular IL-1α staining in various hematopoietic cell types including hematopoietic progenitors. The results showed that granulocytes, B-cells, T-cells, monocytes and macrophages from Ly6G-Cre *JAK2*^*+/+*^ and *JAK2*^*+/VF*^ mice expressed similar IL-1α protein levels, respectively (Fig. 2D). However, MKP cells from Ly6G-Cre *JAK2*^*+/VF*^ mice displayed significantly higher IL-1α protein levels (Fig. 2D). Together, this data suggests that MKPs are a major source of the elevated IL-1α serum levels in Ly6G-Cre *JAK2*^*+/VF*^ mice. However, non-hematopoietic stromal cells may also participate in this process.

### Cytokine gene expression signatures in *JAK2-V617F* and *CALRmut* neutrophils isolated from mice and humans

**Fig. 3:**
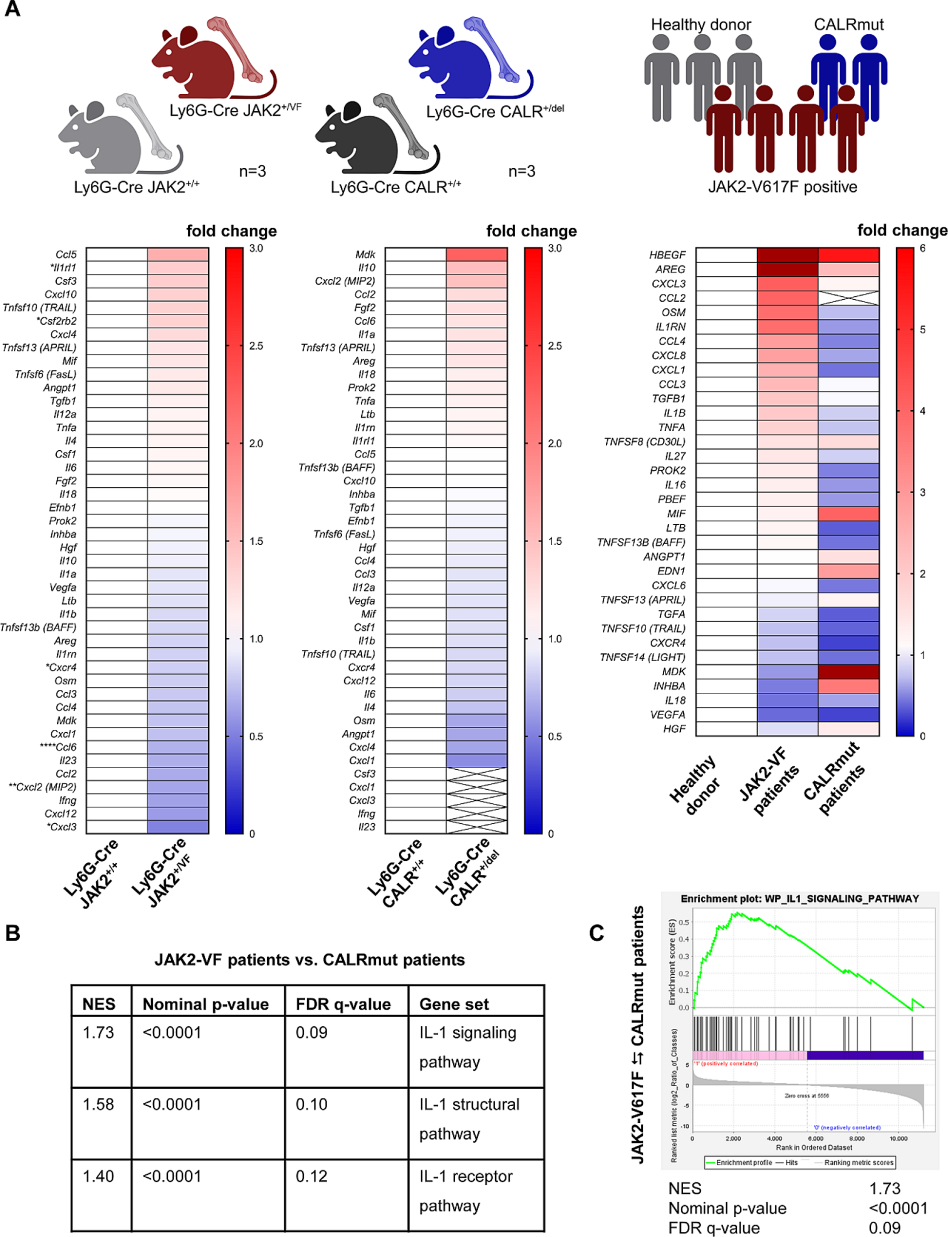
RNA-seq in granulocytes from *JAK2-V617F* and *CALR-mutated* mice and patients shows distinct inflammatory cytokine signatures (**A**) Left and middle panel: RNA-seq of granulocytes obtained from Ly6G-Cre *JAK2*^*+/+*^, *JAK2*^*+/VF*^, *CALR*^*+/+*^ and *CALR*^*+/del*^ mice (each *n* = 3) was performed by GENEWIZ Inc. (Leipzig, Germany). Using DESeq2, a comparison of gene expression between the *JAK2*^*+/+*^ versus *JAK2*^*+/VF*^ and *CALR*^*+/+*^ versus *CALR*^*+/del*^ groups of samples was performed. The heatmaps represent fold changes in mean normalized counts of cytokine RNA abundances relative to the WT controls. Left panel: Ly6G-Cre *JAK2*^*+/+*^ versus *JAK2*^*+/VF*^ mice; middle panel: Ly6G-Cre *CALR*^*+/+*^ versus *CALR*^*+/del*^ mice. *adjusted p-value ≤ 0.05, **adjusted p-value ≤ 0.01, ****adjusted p-value ≤ 0.0001; right panel: RNA-seq was performed on peripheral blood granulocytes isolated from *JAK2-V617F* positive (*n* = 4), healthy donors (*n* = 3) and *CALR-mutated* (*n* = 2) patients by GENEWIZ Inc. (Leipzig, Germany). Using DESeq2, a comparison of RNA expression between JAK2-V617F positive patients versus age-matched healthy donors and CALR-mutated patients versus healthy donors was performed. The heatmaps depict fold changes of mean normalized counts of cytokine RNA abundances relative to the healthy donor controls. Cartoon created with Biorender.com. (**B**) GSEA of IL-1 pathways in granulocytes isolated from *JAK2-V617F* positive patients compared to *CALR-mutated* patients. Positive NES in the heatmap indicate substantial (FDR q-values < 0.15) enrichment in granulocytes from *JAK2-V617F* positive patients. The values point out NES for each pathway. All gene sets were obtained from the GSEA website (UC San Diego and Broad Institute; https://www.gsea-msigdb.org). (**C**) RNA-seq data from *JAK2-V617F* positive patients (*n* = 4 consecutive patients) compared to CALR-mutated (*n* = 2 consecutive patients) patients is tested for enrichment of genes related to the “Interleukin 1 Signaling Pathway” by Gene Set Enrichment Analysis (GSEA). GSEA was performed using the GSEAv4.3.2 (UC San Diego and Broad Institute; https://www.gsea-msigdb.org). Comparisons exhibiting a p-value < 0.05 and FDR q-value < 0.15 were considered significant. The enrichment map was used for visualization of the GSEA results. Normalized Enrichment Score (NES) and False Discovery Rate (FDR) p-values were calculated upon 10,000 gene set permutations

Next, we performed RNA-seq analysis on neutrophils isolated from Ly6G-Cre *JAK2*^*+/+*^, *JAK2*^*+/VF*^, *CALR*^*+/+*^, and *CALR*^*+/del*^ mice. In JAK2-V617 neutrophils, 53 genes out of a total of 14,923 genes analysed were significantly (adjusted p-value < 0.05) regulated (Additional File 1, Tab. S6), whereas in CALRdel granulocytes no significant alteration were noted (data not shown). In order to determine whether the elevated cytokines observed in the serum of JAK2-V617F mice originated from JAK2-V617F granulocytes, the gene expression of a cluster of neutrophil-derived cytokines, previously reported by C. Tecchio, was examined [57]. The heatmap of relative changes induced by JAK2-V617F is depicted in Fig. 3A, left panel. Notably, four chemokine genes (*Cxcl2, Cxcr4, Cxcl3* and *Ccl6*) showed a statistically significant change (adjusted p-value < 0.05), whereas this signature was not present in neutrophils from *CALR*^*+/del*^ mice (Fig. 3A, middle panel). In addition, *Il1rl1* and *Csf2rb2* genes were also significantly (adjusted p-value < 0.05) regulated by JAK2-V617F, but not by CALRdel. This data suggests that the up-regulation of inflammatory cytokines in serum of Ly6G-Cre *JAK2*^*+/VF*^ mice (IL-1α, IL-12 (p40), and M-CSF) does not derive directly from JAK2-V617F neutrophils. Instead, indirect mechanisms are involved employing other hematopoietic and possibly non-hematopoietic cells. An example for this is the IL-1α production in MKPs illustrated in Fig. 2D. Potentially this is regulated via signals from the above mentioned chemokine signature (*Cxcl2, Cxcr4, Cxcl3* and *Ccl6*), which needs to be further investigated. Regarding the mechanism of action of CALRdel in neutrophils, it is worth mentioning that polymorphonuclear cells (PMN) were reported to express low levels of the TPO receptor c-Mpl and that TPO transiently induces low level STAT1 tyrosine phosphorylation in granulocytes [58]. Additionally, Terada and colleagues [59] found that TPO stimulates ex vivo expansion of neutrophils during the early stages of differentiation. Thus, the available literature suggests that neutrophils or a fraction of neutrophils possess functional MPL, indicating that mutated CALR may bind to MPL thereby exhibiting its oncogenic functions. Therefore, we investigated MPL expression on BM-derived neutrophils in our CALRmut model. FACS analysis of BM-derived neutrophils of Ly6G-Cre *CALR*^*+/+*^ and *CALR*^*+/del*^ mice detected low level MPL expression (Additional file 3: Fig. S9A). Furthermore, following incubation with TPO, a slight increase of p-STAT5 was observed in the neutrophils of Ly6G-Cre *CALR*^*+/del*^ mice (Additional file 3: Fig. S9B). However, CALRdel is able to activate the G-CSFR as demonstrated by Chachoua et al. [8]. Therefore, CALRdel may dysregulate intrinsically some processes in granulocytes via its action on G-CSFR.

In order to compare cytokine gene expression in granulocytes obtained from healthy donors with JAK2-V617F and CALR mutant patients (clinical characteristics are depicted in Additional file 1: Tab. S4), we performed RNA-seq and generated a heatmap of cytokine gene expression in human neutrophils (Fig. 3A, right panel). Apparently, the fold-changes were more prominent as compared to the Ly6G-Cre mouse models (Fig. 3A, left and middle panel). However, when considering the adjusted p-values the changes in gene expression were not statistically significant. This is not unexpected, because there is high inter-individual variability which is influenced, among other factors, by inherited genetic predisposition factors and disease phenotype. Nevertheless, the overall observed changes high-lighted differences between the two groups of patients investigated (Fig. 3A, right panel): generally, JAK2-V617F positive patients displayed a greater number of upregulated cytokines in comparison to those carrying CALR mutations, where more cytokines were downregulated. Interestingly, in comparison to healthy donors *CXCL3*, *CCL2*, *CCL4*, *CXCL8*, *CXCL1*, and *CCL3* showed up-regulation in JAK2-V617F positive patients, whereas these genes were unchanged or down-regulated in CALR mutant patients (Fig. 3A, right panel). Furthermore, gene expression of *IL1RN*, *IL1B*, and *TNFA* also displayed up-regulation in JAK2-V617F patients, whereas in CALR-mutant patients they showed a tendency to decrease (Fig. 3A, right panel). This data is interesting, given the crucial role of IL-1β in the development of JAK2-V617F positive disease [17, 18]. However, it requires further analysis in a larger cohort of patients.

To investigate whether we could differentiate between JAK2-V617F and CALR mutant patients based on gene expression profiling, we aimed to determine if there were any differentially expressed functional pathways. We hypothesized that IL-1 signaling could be an interesting candidate based on previously published results [17, 18] and on the increase in gene expression of *IL1B* in neutrophils of JAK2-V617F positive patients (Fig. 3A, right panel). Importantly, Gene Set Enrichment Analysis (GSEA) [60, 61] revealed a remarkable and highly significant enrichment of the “Interleukin 1 Signaling Pathway”, “IL-1 Structural Pathway” and “IL-1R Pathway” in neutrophils sampled from patients with JAK2-V617F, when compared with CALR-mutated patients (Fig. 3B and C). Analysis of JAK2-V617F positive patients versus healthy donors also indicated a trend towards an enrichment of these pathways (Additional file 1: Tab. S5). Together, this data portrays a marked influence of the JAK2-V617F mutation in generating an IL-1 response profile in granulocytes of MPN patients.

### Pan-hematopoietic expression of *JAK2-V617F* induces a prominent inflammatory cytokine signature in neutrophils

**Fig. 4:**
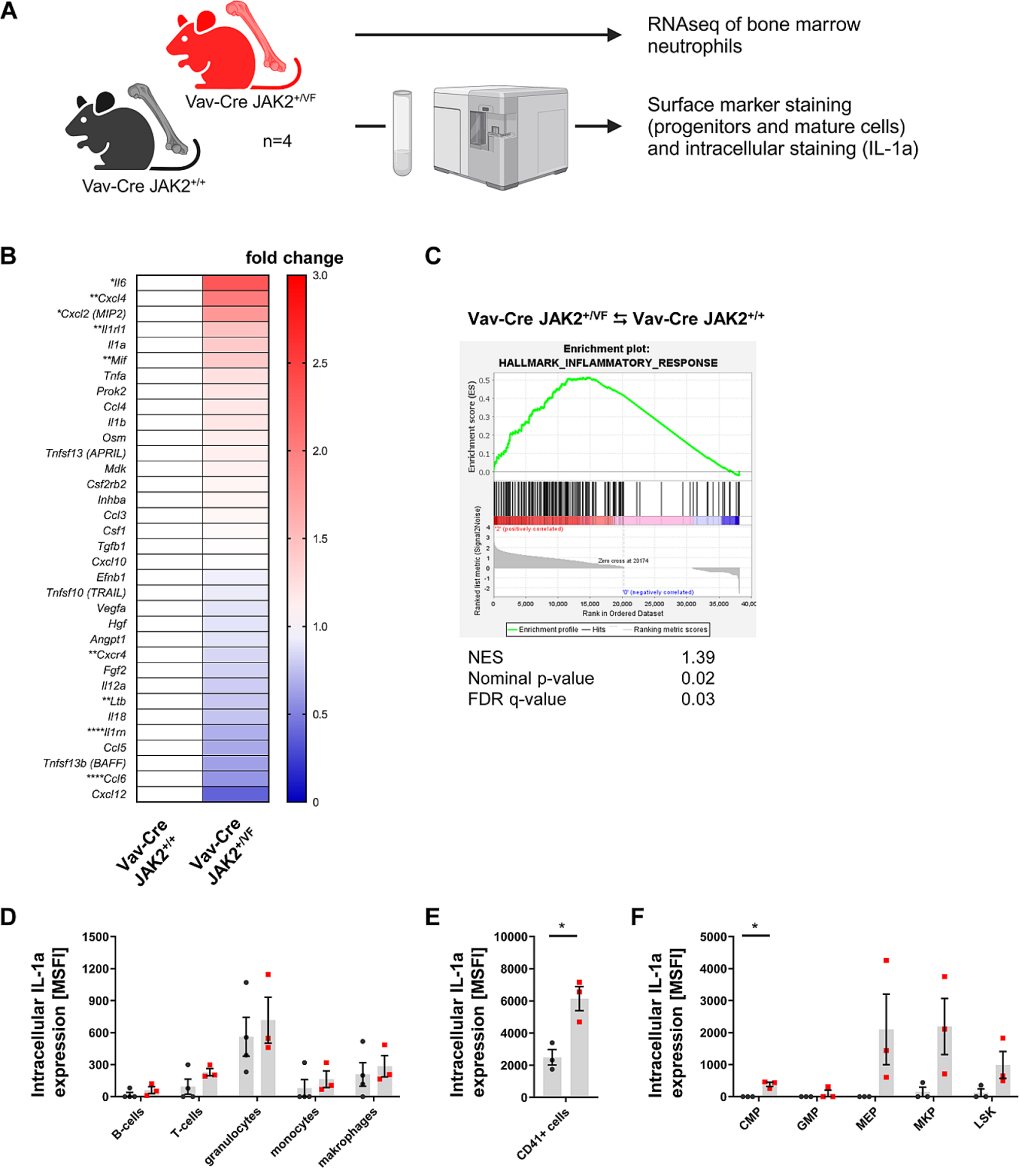
Pan-hematopoietic expression of *JAK2-V617F* up-regulates pro-inflammatory cytokines in neutrophils and hematopoietic progenitors (**A**) Cartoon depicting the experimental design to study gene expression signatures in neutrophils and intracellular IL-1α expression in Vav-Cre *JAK2*^*+/VF*^ mice in comparison to their corresponding WT controls. Created with Biorender.com. (**B**) RNA-seq of granulocytes obtained from Vav-Cre *JAK2*^*+/VF*^ and *JAK2*^*+/+*^ mice (each *n* = 4) was performed by GENEWIZ Inc. (Leipzig, Germany). Using DESeq2, a comparison of gene expression between the Vav-Cre *JAK2*^*+/+*^ versus *JAK2*^*+/VF*^ groups of samples was performed. The heatmaps represent fold changes in mean normalized counts of cytokine RNA abundances relative to the WT controls. Vav-Cre *JAK2*^*+/+*^ versus *JAK2*^*+/VF*^ mice; *adjusted p-value ≤ 0.05, **adjusted p-value ≤ 0.01, ****adjusted p-value ≤ 0.0001 (**C**) GSEA of “Hallmark Inflammatory Response” genes in granulocytes isolated from Vav-Cre *JAK2*^*+/VF*^ and *JAK2*^*+/+*^ mice. The enrichment map was used for visualization of the GSEA results. Normalized Enrichment Score (NES) and False Discovery Rate (FDR) p-values were calculated upon 10,000 gene set permutations. The positive NES of 1.39 in the figure indicates substantial (FDR q-value = 0.03; nominal p-value = 0.02) enrichment in genes linked with the inflammatory response. The “Hallmark Inflammatory Response” gene set was obtained from the GSEA website (UC San Diego and Broad Institute; https://www.gsea-msigdb.org). (**D, E, F**) Intracellular staining for IL-1α levels in various hematopoietic cell populations using anti-IL-1α antibody and isotype control antibody, respectively was performed as described under Supplemental Methods. Mean fluorescence intensity (MFI) was measured by flow cytometry. The specific MFI (MSFI) was calculated by subtracting the MFI of the isotype control from the MFI of the anti-IL-1α antibody stained sample. Data are shown as mean ± SEM. *p ≤ 0.05 (unpaired, two-tailed t-test with Welch correction)

Unlike in the Ly6G-Cre *JAK2*^*+/VF*^ model, granulocytes in Vav-Cre *JAK2*^*+/VF*^ mice interact with signals from various other JAK2-V617F positive cell populations (such as progenitor cells, MKs, endothelial cells, etc.). This interaction potentially leads to more significant differences in gene expression, compared to the more subtle changes seen in granulocytes from Ly6G-Cre *JAK2*^*+/VF*^ mice. The Vav-Cre *JAK2*^*+/VF*^ mouse model has been previously described and reliably recapitulates a PV-like phenotype of MPN [40, 43]. Neutrophils from Vav-Cre *JAK2*^*+/VF*^ and *JAK2*^*+/+*^ mice were isolated and RNA-seq was performed (Fig. 4A). 3,508 genes out of a total of 13,881 genes were significantly (adjusted p-value < 0.05) regulated in neutrophils from Vav-Cre *JAK2*^*+/VF*^ mice (data not shown). In comparison to the low number of regulated genes in the Ly6G-Cre *JAK2*^*+/VF*^ model, this indicates that the gene expression signature of neutrophils in JAK2-V617F induced disease is not primarily regulated by cell-intrinsic JAK2-V617F expression. Instead, JAK2-V617F neutrophils are more sensitive to extrinsic signals originating from other JAK2-V617F positive hematopoietic cells.

Next, we focused on the cytokine gene expression levels induced by JAK2-V617F and generated a heatmap of relative changes (Fig. 4B). Significant changes (adjusted p-values < 0.05) were detected in nine genes by DESeq2 analysis. *Il6* (2.3-fold change), *Cxcl4* (2.0-fold change), *Cxcl2* (1.8-fold change), *Il1rl1* (1.4-fold change) and *Mif* (1.4-fold change) were identified as the five up-regulated cytokine genes in JAK2-V617F positive neutrophils, while *Cxcr4* (0.8-fold change), *Ltb* (0.7-fold change), *Il1rn* (0.6-fold change) and *Ccl6* (0.58-fold change) showed a decrease. These changes in gene expression did not overlap with the panel of upregulated cytokines in serum of Vav-Cre *JAK2*^*+/VF*^ mice which comprises CCL2, CCL11, CXCL5, CXCL9, CXCL10 and IL-1α (as published previously [43]), again suggesting that neutrophils are not the primary cellular source of upregulated serum cytokines in JAK2-V617F induced disease.

Since neutrophils are recognized as an important pathogenic link to inflammation we performed Gene Set Enrichment Analysis (GSEA) on RNA-seq data from neutrophils of Vav-Cre *JAK2*^*+/VF*^ and *JAK2*^*+/+*^ mice utilizing the “Hallmark Inflammatory Response” signature from the Molecular Signature Database (MSigDB) hallmark gene set collection [62] (Fig. 4C). GSEA indicated a highly significant enrichment of genes linked to “Hallmark Inflammatory Response” in neutrophils from Vav-Cre *JAK2*^*+/VF*^ mice (Fig. 4C). Of note, this signature was not enriched in neutrophils obtained from Ly6G-Cre *JAK2*^*+/VF*^ mice (data not shown). Based on this data, we conclude that the inflammatory gene expression signature of JAK2-V617F neutrophils is particularly prone to external signals originating from other JAK2-V617F positive cells.

Next, we examined which hematopoietic cell populations are the source of the elevated serum levels of inflammatory cytokines in the Vav-Cre *JAK2-V617F* model. We focused on IL-1α (upregulated by a factor of 4.8 in serum of Vav-Cre *JAK2*^*+/VF*^ mice) and performed intracellular IL-1α staining in various hematopoietic cell types including hematopoietic progenitors. The results showed that granulocytes, B-cells, T-cells, monocytes and macrophages from Vav-Cre *JAK2*^*+/+*^ and *JAK2*^*+/VF*^ mice expressed similar IL-1α protein levels, respectively (Fig. 4D). However, CD41^+^ cells from Vav-Cre *JAK2*^*+/VF*^ mice displayed significantly higher IL-1α protein levels (Fig. 4E). Elevated IL-1α levels were also found in CMP (Fig. 4F). MEP, MKP and LSK cells from Vav-Cre *JAK2*^*+/VF*^ mice also trended in this direction (Fig. 4F). Together, this data suggests that MKs and myeloid progenitors but not granulocytes are major contributors to the elevated IL-1α serum levels in Vav-Cre *JAK2*^*+/VF*^ mice. However, in myelofibrosis, it has been shown that both, myeloid progenitors and mature myeloid cells, may produce inflammatory cytokines, yet with distinct cytokine secretion profiles [63].

### Neutrophil-specific expression of *JAK2-V617F* and *CALRdel*, respectively differentially affects the metabolic activity

**Fig. 5:**
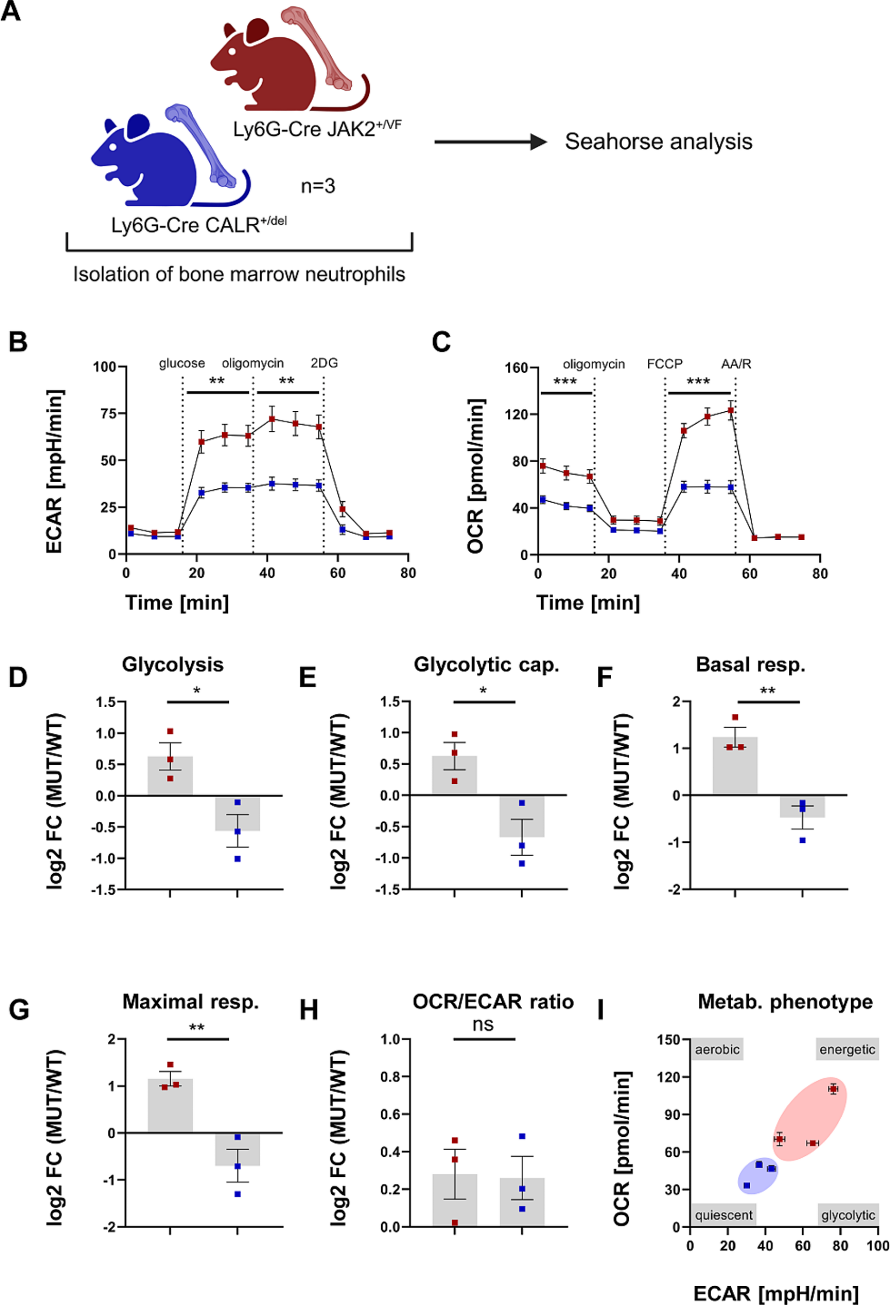
Neutrophils isolated from Ly6G-Cre *JAK2*^*+/VF*^ mice display enhanced metabolic activity. (**A**) Cartoon depicting the experimental design to study metabolic activity in neutrophils isolated from Ly6G-Cre *JAK2*^*+/VF*^ and *CALR*^*+/del*^. Created with Biorender.com. (**B**) Glycolysis stress test (GST) and (**C**) mitochondrial stress test (MST) analyzing extracellular acidification rate (ECAR), as a surrogate for aerobic glycolysis, and oxygen consumption rate (OCR), as a surrogate for oxidative phosphorylation in neutrophils isolated from Ly6G-Cre *JAK2*^*+/VF*^ and Ly6G-Cre *CALR*^*+/del*^ (*n* = 3 with 3–6 technical replicates) were recorded in real-time upon sequential injection of compounds/inhibitors as described in the Supplemental Methods. The data presented were normalized to the background (phase 1 for GST and phase 4 for MST). Based on these and the corresponding wild-type data (Supplemental Fig. 7A and B), the metabolic parameters glycolysis (**D**), glycolytic capacity (**E**), basal respiration (**F**), maximal respiration (**G**), and the OCR/ECAR ratio as a surrogate for the balance between glycolysis and OXPHOS (**H**) were calculated and presented as the log2 fold change (log2 FC) of each individual relative to the respective wild-type average. (**I**) The baseline overall metabolic phenotype was calculated from (**B** and **C**) and plotted as an ECAR/OCR map. Data are shown as mean ± SEM. *p ≤ 0.05, **p ≤ 0.01, ***p ≤ 0.001 (unpaired, two-tailed t-test). Abbreviations: OCR, oxygen consumption rate; ECAR, extracellular acidification rate; 2DG, 2-deoxyglucose; FCCP, Carbonyl cyanide-p-trifluoromethoxyphenylhydrazone; AA/R, Antimycin A/Rotenone

It is well established that an inflammatory phenotype of immune and tissue-resident cells is reflected by, if not dependent on, an altered cellular metabolism for meeting the cells’ increased metabolic demands [45, 46]. Therefore, we compared the metabolic profile of neutrophils isolated from Ly6G-Cre *JAK2*^*+/VF*^ and *CALR*^*+/del*^ mice by means of real-time metabolic flux analyses (Fig. 5A). Neutrophils from *JAK2*^*+/VF*^ mice displayed an overall increased metabolic activity (Fig. 5B-G). Both the glycolytic (Fig. 5B) as well as the respiratory (Fig. 5C) parameters tested were significantly higher compared to the neutrophils isolated from Ly6G-Cre *CALR*^*+/del*^ mice. When compared to the respective wild type controls (Additional file 3: Fig. S10A and B), the metabolic parameters glycolysis (Fig. 5D), glycolytic capacity (Fig. 5E), basal respiration (Fig. 5F), maximal respiration (Fig. 5G), revealed higher activity in JAK2-V617F neutrophils and less activity in CALRdel neutrophils. Additional metabolic parameters are depicted in Additional file 3: Fig. S10 and are in line with these results. The OCR/ECAR ratio (Fig. 5H) as a surrogate for the balance between glycolysis and OXPHOS was similarly increased in both genotypes. However, the baseline overall metabolic phenotype (Fig. 5I) plotted as an ECAR/OCR map displayed a clear segregation between JAK2-V617F and CALRdel neutrophils with a more energetic profile for the former and a more quiescent profile for the latter. Thus, it appears that the higher inflammatory activity of JAK2-V617F neutrophils as characterized by the marked pro-inflammatory cytokine profiles described above is mirrored by a striking increase in the metabolic activity. It will be interesting to test this hypothesis in future studies by using inhibition experiments. Additional metabolic parameters including comparisons of the respective WT controls and the comparisons between JAK2-V617F versus WT and CALRdel versus WT are shown in Additional file 3: Fig. S10.

### Neutrophil-specific expression of *JAK2-V617F*, but not *CALRdel*, decreases migration of granulocytes in vitro

**Fig. 6:**
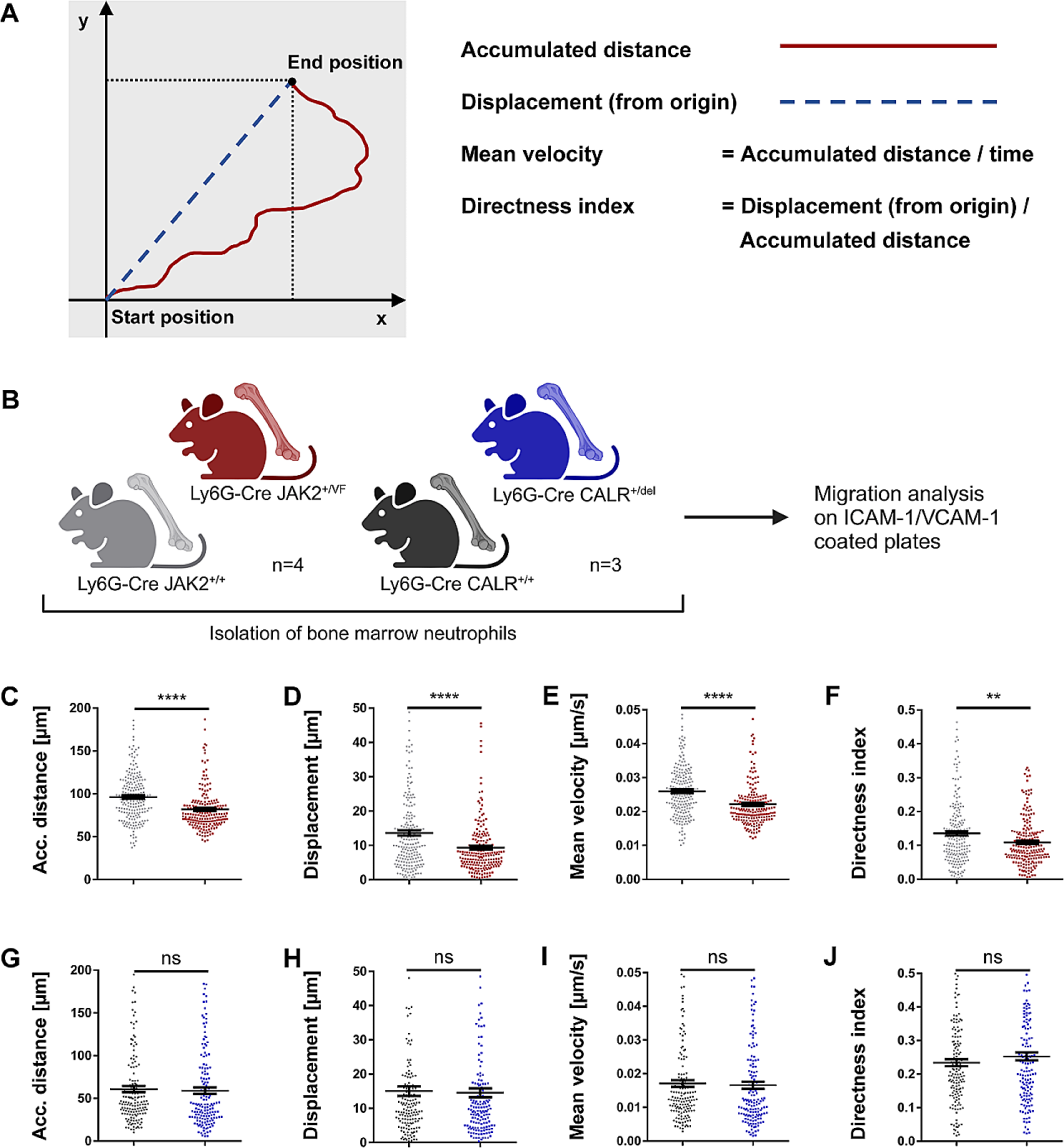
Neutrophil-specific JAK2-V617F, but not CALRdel, changes the migration behavior of neutrophils in vitro. (**A**) Scheme of cell migration parameters investigated. (adapted from Zengel et al.; [68]. Accumulated distance from origin to end position is indicated as a red line. Displacement as minimum distance between origin and end position is depicted with the blue dashed line. Mean velocity is calculated from the ratio of accumulated distance and time. Directness as ratio of displacement and accumulated distance indicates a measure of how direct a cell migrates from its origin to its end position. A directness value tending towards 1 indicates a straight migration from the start to the end position. Thus, an increase in directness index indicates that cells more directly migrate from their origin to their end position. (**B**) Cartoon depicting the experimental design to study migration characteristics in neutrophils isolated from Ly6G-Cre *JAK2*^*+/VF*^ and *CALR*^*+/del*^ mice in comparison to their corresponding WT controls. Created with Biorender.com. (**C-F**) Microfluidic chambers were coated with recombinant mouse ICAM-1 and VCAM-1. tdTomato expressing neutrophils isolated from Ly6G-Cre *JAK2*^*+/VF*^ and *JAK2*^*+/+*^ mice were seeded and time lapse recording of cell migration was started. Neutrophils isolated (200 tdTomato^+^ neutrophils investigated) from Ly6G-Cre *JAK2*^*+/+*^ and *JAK2*^*+/VF*^ mice (each *n* = 4) exhibited the following migration parameters: accumulated distance of 96.2 μm compared to 81.9 μm, displacement of 13.6 μm compared to 9.3 μm, mean velocity of 0.026 μm/sec compared to 0.022 μm/sec, and directness index of 0.14 compared to 0.11. (**G-J**) Migration parameters obtained in 150 tdTomato^+^ neutrophils investigated in Ly6G-Cre *CALR*^*+/+*^ and *CALR*^*+/del*^ mice (each *n* = 3). Data are shown as mean ± SEM. **p ≤ 0.01, ****p ≤ 0.0001 (unpaired, two-tailed t-test)

Inflammatory conditions cause granulocytes to be sequestered from the circulation and directed towards the endothelial layer of blood vessels via the leukocyte adhesion cascade process [64, 65]. Cell migration of granulocytes on the endothelium is a multi-faceted biological process that relies heavily on molecular communication between integrin receptors LFA-1 and VLA-4 and their corresponding ligands, ICAM-1 and VCAM-1, along with integrin outside-in signaling and cytoskeletal re-organization that prompts actin polymerization (for reviews, see Guenther-C [65], De Pascalis & Etienne-Manneville [66] and Nourshargh & Alon [67]). Both VLA-4 - VCAM-1 and LFA-1 - ICAM-1 interactions play significant roles in regulating neutrophil adhesion and leukocyte-vessel wall interactions [67]. Therefore, we investigated cell migration of granulocytes on VCAM-1 and ICAM-1 coated surfaces (Fig. 6A). To accurately quantify cell movement, we considered the parameters which are visually represented in Fig. 6B: accumulated distance, displacement from origin, mean velocity (speed) and directness of migration (directness index). Neutrophils obtained from Ly6G-Cre *JAK2*^*+/VF*^ mice demonstrated a significant decrease in both accumulated distance (85% of WT control) and displacement from origin (68% of WT control) as shown in Fig. 6, C and D and in Additional file 1: Tab. S7. Significantly, no notable differences in these parameters were found in *CALR*^*+/del*^ mice (Fig. 6, G and H and Additional file 1: Tab. S6). Additionally, the mean velocity of JAK2-V617F positive neutrophils was noticeably lower (at 85% of WT control) (Fig. 6E and Additional file 1: Tab. S7), whereas no variations were observed in the CALRdel model (Fig. 6I and Additional file 1: Tab. S7). Finally, the mean directness index indicating the directed migration of JAK2-V617F positive neutrophils from their origin to their final position was diminished to 79% of the corresponding WT control group (Fig. 6F and Additional file 1: Tab. S7). Again, no significant differences were observed in CALRdel compared to CALR-WT neutrophils (Fig. 6J and Additional file 1: Tab. S7). These data indicate a significant decrease in the migratory capacity of neutrophils expressing JAK2-V617F, but not of those expressing CALRdel. As a consequence, this may translate into pronounced accumulation of JAK2-V617F neutrophils at the site of an inflamed environment in vivo.

### Partial ligation of the saphenous vein demonstrates reduced migration of *JAK2-V617F* positive neutrophils in vivo

**Fig. 7:**
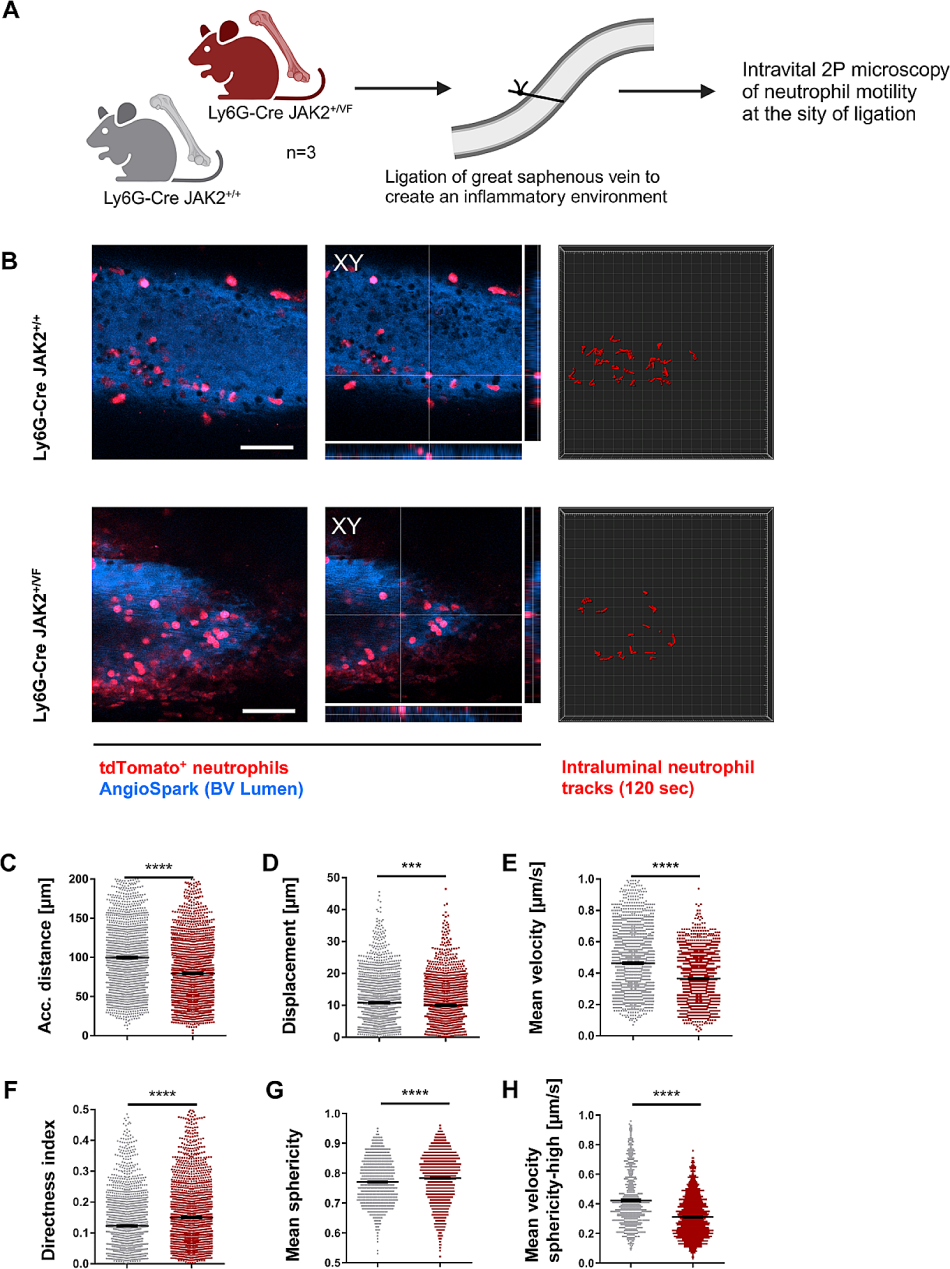
*JAK2-V617F* suppresses the motility of neutrophils in an inflammatory environment. (**A**) Cartoon depicts experimental design to investigate neutrophil intraluminal crawling after partial ligation of the great saphenous vein (GSV) using intravital 2P microscopy. Created with Biorender.com. (**B**) To identify neutrophils (red) within the GSV from those localized in the surrounding connective tissue, AngioSPARK680 (blue) was applied intravenously. Imaging was started 20 min after applying partial ligation of the GSV, two examples from imaging after 80 min are shown for Ly6G-Cre *JAK2*^*+/+*^ (upper panels) and *JAK2*^*+/VF*^ (lower panels) are shown. Left: Image projection of tree z-planes spanning 15 μm. Scale bar, 50 μm. Middle: XYZ sections of an example neutrophil on the vessel wall. Right: Neutrophil motion tracks of 120 s of imaging each. (**C-H**) Measurements of in vivo two-photon microscopy showing accumulated distance, displacement, mean velocity, directness index, and mean sphericity of 2,339 tdTomato^+^ neutrophils from Ly6G-Cre *JAK2*^*+/+*^ and 2,334 tdTomato^+^ neutrophils from Ly6G-Cre *JAK2*^*+/VF*^ mice (*n* = 3 per condition) as well as the mean velocity of sphericity^high^ neutrophils. Neutrophils isolated exhibited the following migration parameters (*JAK2*^*+/+*^ compared to *JAK2*^*+/VF*^): accumulated distance of 99.7 μm compared to 79.4 μm, displacement of 10.8 μm compared to 10.0 μm, mean velocity of 0.464 μm/sec compared to 0.365 μm/sec, and directness index of 0.12 compared to 0.15. Data are shown as mean ± SEM. ***p ≤ 0.001, ****p ≤ 0.0001 (unpaired, two-tailed t-test)

To examine neutrophil migration on the endothelium in an inflamed vessel in vivo, we utilized intravital 2P microscopy of neutrophils in Ly6G-Cre *JAK2*^*+/VF*^ mice after partially ligating the great saphenous vein (GSV) which provokes an inflammatory condition (Fig. 7A and B). Notably, we found a general decline of migration parameters in line with the in vitro observation: a decrease in the accumulated distance to 80% and a decrease in the mean displacement to 93% of the WT controls in the detected tracks of JAK2-V617F positive neutrophils (Fig. 7, C and D and Additional file 1: Tab. S7).

Additionally, JAK2-V617F neutrophils migrated significantly slower than JAK2-WT cells, with a mean velocity reduced to 79% of the WT control (Fig. 7E and Additional file 1: Tab. S7). However, the mean directness index of JAK2-V617F positive neutrophils in vivo was higher (125%) as compared to the WT group (Fig. 7F and Additional file 1: Tab. S7). The increased directness index indicates that despite their slower speed, JAK2-V617F neutrophils migrate in a more direct manner from their origin to their end position across the endothelium of an inflamed vessel. Moreover, we observed that cell sphericity in the JAK2-V617F neutrophils was higher than in the WT controls, suggesting enhanced rolling and adhesion of intravascular cells at the endothelium (Fig. 7G). Analysis of the only cell fraction with high sphericity (i.e. above the median sphericity of all tracks) showed a strong decrease in mean velocity in the JAK2-V617F neutrophils, indicating that these cells were rolling slower on the vascular endothelium than WT controls (Fig. 7H). Therefore, increased rolling and tethering, reduced accumulated distance and enhanced directness of cell movement in vivo may result in increased gathering of JAK2-V617F positive neutrophils at the inflamed endothelium site, ultimately contributing to the pro-thrombotic state of JAK2-V617F positive disease. Using intravital 2P-microscopy, we have shown previously [69] that inhibition of integrin VLA4 expressed on granulocytes strongly impacts the mobility of granulocytes in vessels.

### Expression of *JAK2-V617F* and *CALRdel*, respectively re-programs the binding characteristics to ICAM-1, VCAM-1 and selectins in neutrophils

**Fig. 8:**
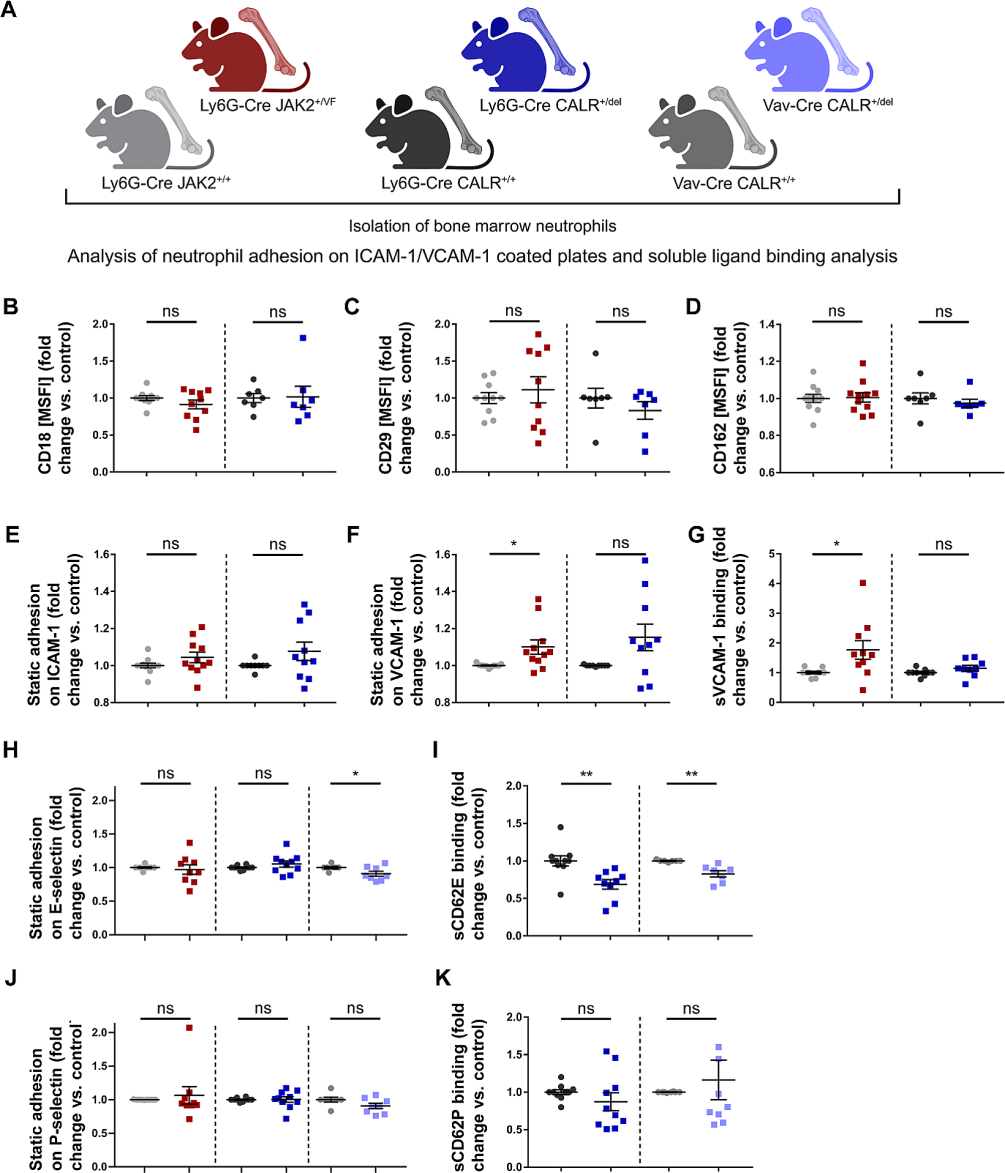
Adhesion and soluble ligand binding characteristic of JAK2-V617F and CALRdel-expressing neutrophils (**A**) Cartoon depicting the experimental design to study adhesion characteristics in neutrophils isolated from Ly6G-Cre *JAK2*^*+/VF*^ and *CALR*^*+/del*^ mice and from Vav-Cre *CALR*^*+/del*^ mice in comparison to their corresponding WT controls. Created with Biorender.com. (**B-D**) Expression of CD18 (β2 integrin), CD29 (β1 integrin) and CD162 (PSGL-1) was evaluated as MSFI and calculated as fold change versus control. (**B, C**) Expression of CD18 (β2 integrin) and CD29 (β1 integrin) of tdTomato^+^ cells of Ly6G-Cre *JAK2*^*+/+*^ (*n* = 10), *JAK2*^*+/VF*^ (*n* = 10), *CALR*^*+/+*^ (*n* = 7) and *CALR*^*+/del*^ mice (*n* = 7). (**D**) Expression of CD162 (PSGL-1) of tdTomato^+^ cells of Ly6G-Cre *JAK2*^*+/+*^ (*n* = 11), *JAK2*^*+/VF*^ (*n* = 11), *CALR*^*+/+*^ (*n* = 7) and *CALR*^*+/del*^ mice (*n* = 7). (**E, F**) Static adhesion of tdTomato^+^ cells isolated from *JAK2*^*+/+*^ (*n* = 11), *JAK2*^*+/VF*^ (*n* = 11), *CALR*^*+/+*^ (*n* = 9) and *CALR*^*+/del*^ mice (*n* = 10) on Fc-free ICAM-1 and VCAM-1 coated plates. Static adhesion assays revealed significant differences in tdTomato^+^ neutrophil adhesion to VCAM-1 between *JAK2*^*+/+*^ and *JAK2*^*+/VF*^ mice (JAK2-V617F: 1.1 ± 0.038; WT: 1.0 ± 0.003; *p* = 0.0163; *n* = 11). No differences were shown in static adhesion to ICAM-1. (**G**) Soluble ligand binding to Fc-tagged VCAM-1 of tdTomato^+^ cells isolated from *JAK2*^*+/+*^ (*n* = 10), *JAK2*^*+/VF*^ (*n* = 10), *CALR*^*+/+*^ (*n* = 9) and *CALR*^*+/del*^ mice (*n* = 9). Fold change versus control analysis. Soluble ligand binding to VCAM-1/Fc was increased and significantly higher in tdTomato^+^ neutrophils of *JAK2*^*+/VF*^ mice compared to their corresponding WT control (1.77 ± 0.312; WT: 1.0 ± 0.044; *p* = 0.0258; *n* = 10). *p ≤ 0.05 (unpaired, two-tailed t-test). (**H**) Left and middle panel: Static adhesion of sorted tdTomato^+^ neutrophils isolated from *JAK2*^*+/VF*^ (*n* = 9) and *CALR*^*+/del*^ (*n* = 10) mice to Fc-free E-selectin coated plates in comparison to their corresponding *JAK2*^*+/+*^ (*n* = 9) and *CALR*^*+/+*^ (*n* = 10) controls. Right panel: Neutrophils harvested from Vav-Cre *CALR*^*+/del*^ mice (*n* = 8) showed significantly impaired adhesion to immobilized E-selectin compared to Vav-Cre *CALR*^*+/+*^ (*n* = 7) mice (0.91 ± 0.036; WT: 1.00 ± 0.016; *p* = 0.0443). (**I**) Binding to soluble Fc-tagged E-selectin of granulocytes isolated from Ly6G-Cre *CALR*^*+/+*^ (*n* = 10), Ly6G-Cre *CALR*^*+/del*^ (*n* = 9) as well as Vav-Cre *CALR*^*+/+*^ (*n* = 7) and Vav-Cre *CALR*^*+/del*^ (*n* = 7) mice, respectively shown as fold change versus control analysis. Binding of tdTomato^+^ granulocytes isolated from Ly6G-Cre *CALR*^*+/del*^ mice to soluble E-selectin was found significantly decreased compared to Ly6G-Cre *CALR*^*+/+*^ mice (0.69 ± 0.063; WT: 1.00 ± 0.068; *p* = 0.0039). Binding to soluble Fc-tagged E-selectin (sCD62E) of bone marrow granulocytes derived from Vav-Cre *CALR*^*+/del*^ mice was also found significantly reduced in comparison to corresponding WT mice (0.83 ± 0.043; WT: 1.00 ± 0.005; *p* = 0.0018). **p ≤ 0.01 (unpaired, two-tailed t-test) Exclusion of one outlier in Ly6G-Cre *CALR*^*+/del*^ and Vav-Cre *CALR*^*+/del*^ mice, respectively with the Grubbs’ outlier test (Ly6G-Cre *CALR*^*+/del*^: G = 2.324, α = 0.05; Vav-Cre *CALR*^*+/del*^: G = 2.271, α = 0.05). (**J**) Left and middle panel: Static adhesion of sorted tdTomato^+^ neutrophils isolated from Ly6G-Cre *JAK2*^*+/VF*^ (*n* = 9) and *CALR*^*+/del*^ (*n* = 10) mice to Fc-free P-selectin coated plates in comparison to their corresponding *JAK2*^*+/+*^ (*n* = 9) and *CALR*^*+/+*^ (*n* = 10) controls. Right panel: Static adhesion of isolated granulocytes from Vav-Cre *CALR*^*+/del*^ (*n* = 8) and *CALR*^*+/+*^ (*n* = 7) mice to Fc-free P-selectin coated plates (0.91 ± 0.04; WT: 1.00 ± 0.037). (**K**) Binding to soluble P-selectin/Fc (sCD62P) of neutrophils isolated from Ly6G-Cre *CALR*^*+/del*^ (*n* = 10) and Vav-Cre *CALR*^*+/del*^ (*n* = 8) mice compared with Ly6G-Cre *CALR*^*+/+*^ (*n* = 10) and Vav-Cre *CALR*^*+/+*^ (*n* = 8) control mice, respectively. Exclusion of one outlier in Vav-Cre *CALR*^*+/del*^ mice with the Grubbs’ outlier test (G = 2.367, α = 0.05). Data are shown as mean ± SEM. *p* ≤ 0.05, **p ≤ 0.01 (unpaired, two-tailed t-test)

Leukocyte migration on the endothelium employs the mesenchymal migration mode and relies heavily on integrins [65]. Previous studies have suggested that increased binding of integrin receptors to their ligands negatively affects cell motility [70, 71]. Therefore, we investigated the activation of β1 and β2 integrins and of selectins in neutrophils derived from Ly6G-Cre *JAK2*^*+/VF*^ and *CALR*^*+/del*^ mice (Fig. 8A). The expression levels of CD18 (β2 integrin chain present in LFA1), CD29 (β1 integrin chain present in VLA4) and CD162 (PSGL-1, which binds to E- and P-selectin) were found to be unchanged (Fig. 8, B-D). On neutrophils, VLA-4 and LFA-1 are the primary β1 and β2 integrins that bind to VCAM-1 and ICAM-1, respectively. Static adhesion of neutrophils from Ly6G-Cre *JAK2*^*+/VF*^ and *CALR*^*+/del*^ mice to ICAM-1-coated plates was comparable to that of their WT counterparts (Fig. 8E). However, tdTomato^+^ neutrophils from Ly6G-Cre *JAK2*^*+/VF*^ mice exhibited a slightly but significantly increased static adhesion to VCAM-1-coated surfaces (Fig. 8F). However, there was a considerable variability with similar patterns for both groups. Nevertheless, differences in adhesion to VCAM-1 coated surfaces between the models (Ly6G-Cre JAK2-V617F, Ly6G-Cre CALRdel) are supported by the increase in affinity of JAK2-V617F neutrophils in the soluble soluble ligand binding assay (sVCAM-1 binding) (Fig. 8G). Here, the neutrophils of Ly6G-Cre *JAK2*^*+/VF*^ mice exhibited significantly greater binding of soluble VCAM-1 (mean fold increase 1.77 ± 0.312) compared to control mice (Fig. 8G). This indicates an increased affinity of the β1 integrin receptor VLA-4 towards VCAM-1 on *JAK2*^*+/VF*^ neutrophils. It further suggests a conformational change of VLA-4 from the closed to the intermediate or open conformation [39]. In contrast to *JAK2*^*+/VF*^ neutrophils, neutrophils derived from CALRdel mice did not display significant changes in either static adhesion to VCAM-1 and ICAM-1 or in soluble ligand binding (Fig. 8, E-G). Taken together, these data demonstrate that JAK2-V617F but not CALRdel alters the integrin function of VLA-4 on neutrophils resulting in a pro-adhesive phenotype. The pro-adhesive VLA4 – VCAM-1 interaction most likely accounts for the reduced motility of JAK2-V617F positive neutrophils shown in Fig. 6 and in vessels which regularly express VCAM-1 (Fig. 7). These data are novel and intriguing given the important role of granulocytic VLA-4 - VCAM-1 binding in thrombosis and the less frequent thrombotic events observed in CALR-mutant patients.

In inflamed tissues, recruitment of granulocytes from the circulation to the endothelial layer is initiated by binding to selectins resulting in low speed rolling of granulocytes on the endothelial layer [72]. Therefore, we next examined the adhesion of neutrophils isolated from Ly6G-Cre *JAK2*^*+/VF*^ and *CALR*^*+/del*^ mice to E-selectin and P-selectin coated plates. For E-selectin, no differences were detected between Ly6G-Cre JAK2-V617F and Ly6G-Cre CALRdel granulocytes compared to their respective WT controls (Fig. 8H, left and middle panels). However, we performed additional experiments using granulocytes isolated from Vav-Cre *CALR*^*+/del*^ mice. Here, granulocytes were exposed in vivo to signals derived from various other CALRdel positive hematopoietic and endothelial cells. Interestingly, neutrophils isolated from Vav-Cre *CALR*^*+/del*^ mice showed a small but significant decrease in static adhesion to E-selectin (Fig. 8H, right panel). These data suggest that in the Vav-Cre *CALR*^*+/del*^ model, signals derived from CALRdel positive cells other than neutrophils induce a decrease in the static adhesion capacity to bind to E-selectin-coated layers. However, it appears that the cell-intrinsic CALRdel signal in neutrophils is sufficient to reduce the binding affinity to soluble E-selectin by 31% in the Ly6G CALRdel model (Fig. 8I, left panel). As this is a relatively strong change and E-selectin is considered to be the most important selectin for cell trafficking to sites of inflammation, we performed additional experiments in the Vav-Cre CALRdel model. Here, the CALRdel-induced decrease in binding affinity for soluble E-selectin was confirmed in granulocytes from Vav-Cre *CALR*^*+/del*^ mice (Fig. 8I, right panel). In contrast to E-selectin, static adhesion to P-selectin-coated plates and binding affinity to soluble P-selectin were similar in neutrophils derived from the three genotypes studies (Fig. 8, J and K). However, the functional implications of the above findings need to be further evaluated in future studies. PSGL-1 is the dominant ligand for P-selectin and also binds E-selectin and as shown above (Fig. 8D) was equally expressed on neutrophils isolated from Ly6G *JAK2*^*+/VF*^ and *CALR*^*+/del*^ mice compared to their respective WT controls. Taken together, these data highlight for the first time that CALRdel52 down-regulates neutrophil binding to E-selectin, but does not affect binding to ICAM-1 and VCAM-1 which are regularly expressed on endothelium. In the clinical context of MPN disease, it is tempting to hypothesize that this may translate into a reduced pro-thrombotic risk of CALR mutant patients in comparison to JAK2-V617F positive patients.

## Discussion

Unexpectedly, neutrophil-specific expression of *JAK2-V617F* in mice revealed thrombocytosis and BM-MK hyperplasia. Interestingly, hyperplasia of BM-MKs is regularly observed in PMF, where it is a diagnostic hallmark. JAK2-V617F positive MKs have been reported to significantly contribute to the MPN phenotype by inducing erythrocytosis, thrombocytosis, splenomegaly and expansion of HSPCs [73, 74]. TPO regulates PLT counts, but TPO serum levels remained unchanged in Ly6G-Cre JAK2-V617F mice. In addition to TPO, various other cytokines including IL-1β, IL-1α, and IL-6 have been identified to regulate both MKs and platelets [75–77]. Our in vitro studies showed that IL-1β indeed stimulates MK differentiation of lin^−^ hematopoietic progenitors. Thus, in context with the recent discoveries that IL-1β plays a pivotal role in JAK2-V617F-induced thrombocytosis and MK hyperplasia [17, 18], our data suggest that upregulation of IL-1β serum levels in Ly6G-Cre *JAK2*^*+/VF*^ mice drives or participates in generating thrombocytosis.

The inflammatory cytokine signature observed in MPN is associated with a number of constitutive symptoms compromising patients’ quality of life [78]. Specific cytokine-phenotype associations and prognostically relevant plasma cytokine signatures in PMF [79] and PV [80] have been reported. Autocrine and paracrine regulation of inflammatory cytokines produced by clonal and non-clonal hematopoietic cells as well as other cell types like endothelial cells and fibroblasts play a significant role in the inflammatory serum cytokine signature [63, 81, 82]. IL-1β plays a pivotal role in chronic inflammation of MPNs, and its connection to JAK2-V617F/CALR mutants, is widely acknowledged. It is believed that IL-1β levels in blood or bone marrow contribute to increased secretion of inflammatory cytokines by monocytes and macrophages [83]. A recent study by Rai et al. demonstrated that the JAK2-mutant hematopoietic cells are involved in IL-1ß production and thereby induce inflammatory environment, which then helps the JAK2-V617F clones to expand and induce the MPN disease [84]. Besides, in another study by Rai et al. [17], the authors observed the beneficial effects of anti-IL1β antibody treatment, alone or combined with ruxolitinib, on myelofibrosis. Additionally, their investigation using an IL-1β knock-out JAK2V617F mouse model revealed that IL-1β knockout led to reduced levels of inflammatory cytokines, consequently decreasing megakaryopoiesis and myelofibrosis. Moreover, research by Allain-Maillet et al. [85] aimed at understanding the impact of JAK2-V617F mutation on cytokine storms found that while 26 cytokines were overexpressed in JAK2-V617F-mutated MPN patients, only IL-1, IL-1R, and IP-10 were directly induced by JAK2-V617F [85]. In contrast to the well-established association of JAK2-V617F with inflammation and cytokine production, evidence regarding CALR mutation is less abundant. In CALR-mutated ET, the only observed increases in cytokines are IL-4, IL-9, and IL-26, which are typically produced by non-mutated T-cells.

In Ly6G-Cre *JAK2*^*+/VF*^ mice, levels of eight cytokines in the serum were observed to be upregulated by more than 1.5-fold including IL-1α, IL-1β, IL-2, IL-10, IL-12p40, IL-17, M-CSF, and TNFα. Interestingly, this panel of cytokines displays a substantial overlap with the upregulated plasma cytokine levels observed in JAK2-V617F positive MF patients [79]. Elevated serum levels of IL-1α were also observed in a small cohort of MPN patients [86] and in MPLW515L-mutant mice [87]. Thus, our data illustrate that JAK2-V617F expression in neutrophils is sufficient to induce a typical MPN pro-inflammatory cytokine profile in the serum. On the contrary, upregulation of pro-inflammatory cytokines was almost absent in serum of Ly6G-Cre *CALR*^*+/del*^ mice. This is in line with the clinical observation of less frequent inflammatory symptoms of CALR mutated MPN patients [26]. Differential expression of TNFα protein in primary hematopoietic cell populations isolated from JAK2-V617F versus CALR mutant patients has also been described by Fisher and colleagues [88].

Employing RNA-seq in neutrophils isolated from Vav-Cre *JAK2*^*+/VF*^ and *JAK2*^*+/+*^ mice, we found a higher number of up-regulated inflammatory cytokines in comparison to Ly6G-Cre *JAK2*^*+/VF*^ mice and overall enrichment in genes linked to inflammatory response. In contrast to neutrophils obtained from the Ly6G-Cre mouse models, these granulocytes had interacted with numerous other JAK2-V617F positive hematopoietic cell populations in vivo. Importantly, on the protein level, the serum cytokine signature of Vav-Cre *JAK2*^*+/VF*^ mice [43] also shows a higher number of significantly up-regulated inflammatory cytokines (IL-1α, CCL2, CCL11, CXCL5, CXCL9, CXCL10 as reported previously [43]) when compared to the serum cytokine profile revealed by Ly6G-Cre *JAK2*^*+/VF*^ mice. Along this line, RNA-seq analysis of granulocytes isolated from JAK2-V617F patients showed marked up-regulation of a number of inflammatory cytokines and GSEA revealed a highly significant enrichment of IL-1 signaling genes compared to CALR-mutated patients. Together, our findings support the concept that JAK2-V617F positive neutrophils, rather than CALR mutant neutrophils, play an important role in driving chronic inflammation in MPN through an indirect mechanism that involves MKs, MKPs and other myeloid progenitors.

Metabolic reprogramming represents a hallmark of cancer cells. Moreover, an inflammatory cell phenotype is accompanied by altered cellular bioenergetics [45, 46]. Remarkably, both the glycolytic as well as the mitochondrial respiratory activity were significantly higher in JAK2-V617F neutrophils compared to CALRdel neutrophils. This is likely driven by autocrine and paracrine inflammatory cytokines highly expressed in Ly6G-Cre *JAK2*^*+/VF*^ mice. Rao and colleagues, although not specifically investigating neutrophils, already reported that JAK2-V617F promotes both glycolysis and mitochondrial respiration [89]. However, a direct comparison of metabolic parameters employing JAK2-V617F and CALRdel expressing neutrophils has not been reported previously.

There are several important pathophysiological angles when studying thrombosis which is a multifactorial process [90–92]. Our study is limited to the role of neutrophils. The migration of neutrophils is a fundamental component of inflammation including thromboinflammation (for reviews see Kraus & Gruber [93] and Bhuria [37]). Although our study touched the subject of thrombosis, it was beyond the scope of our paper to investigate thrombus formation per se. Rather, we focused on inflammatory activities of neutrophils and on the pathophysiologic steps which are initiated by JAK2-V617F and CALRdel granulocytes, respectively immediately prior to formation of thrombosis and thereby promote thrombosis. Although degranulation of neutrophils and NETosis would certainly be interesting to look at and has been shown to be of great importance in MPN [94, 95] we are focussed on the early inflammation-induced in vivo changes in neutrophil activity before the onset of NETosis. Therefore, we studied in vitro and in vivo integrin- and selectin-controlled cell migration of neutrophils. Migration of granulocytes involves a dynamic interplay between adhesion events controlled on one side by integrins and selectins and on the other side by cytoskeletal functions [96, 97]. Exertion of cytoskeletal forces to the extracellular matrix is realized by dynamic interactions of the cytoskeleton with the adhesion molecules [93, 98–100]. Ly6G-Cre mice offer a suitable model for studying the cell-intrinsic impact of JAK2-V617F and of CALRdel on migratory behavior, since any indirect signals derived from JAK2-V617F or CALRdel positive cell lineages other than neutrophils can be excluded. Interestingly, previous work has demonstrated that neutrophils and monocytes moving along and adhering to the endothelium provide the initiating stimulus for thrombosis development [101–103]. Hence, the observed differences in migration and adhesion between JAK2-V617F and CALRdel positive neutrophils aligns with the clinical observation that JAK2-V617F is the primary driving force of the pro-thrombotic risk in MPN whereas mutated CALR seems to have a lesser impact [26, 27].

Finally, we discovered that CALRdel52 negatively regulates cell adhesion of neutrophils to selectins. A distinct pattern of post-translational modifications is necessary for E- and P-selectin binding to PSGL-1 and to the additional E-selectin receptor CD44 [94, 95, 104]. Interestingly, E-selectin inhibition using the E-selectin antagonist GMI-1271 resulted in reduced thrombus formation in venous thrombosis models [105, 106]. A phase I study of GMI-1271 in deep vein thrombosis patients indicated that E-selectin inhibition reduced thrombus size without elevated risk of hemorrhage [107]. Thus, reduced adhesion of CALRdel52 mutant neutrophils to E-selectin together with no change in adhesion to VCAM-1 offer novel insights into the pathophysiological mechanisms underlying the less elevated thrombotic risk in CALR mutant patients as compared to JAK2-V617F patients. In their publication, Schürch et al. [108] demonstrated myeloperoxidase deficiency in homozygous CALRdel52 mutations, which was induced by impaired folding of newly synthesized myeloperoxidase due to defective CALR-mediated ER quality control in neutrophils. Considering the central role of CALR in quality control of many glycoproteins, it is tempting to hypothesize that reduced adhesion of CALR^+/del^ neutrophils to E-selectin is the result of altered post-translational modifications of the PSGL-1 receptor. This is in line with the unaltered PSGL-1 levels on neutrophils isolated from Ly6g-Cre CALR^+/del^ mice (Fig. 8), which suggests CALRdel52-induced PSGL-1 dysfunction as a cause of impaired binding affinity to E-selectin, possibly driven by abnormal peptide maturation. Another interesting action of CALR mutants is their effect on calcium flux [109].

## Conclusions

**Fig. 9:**
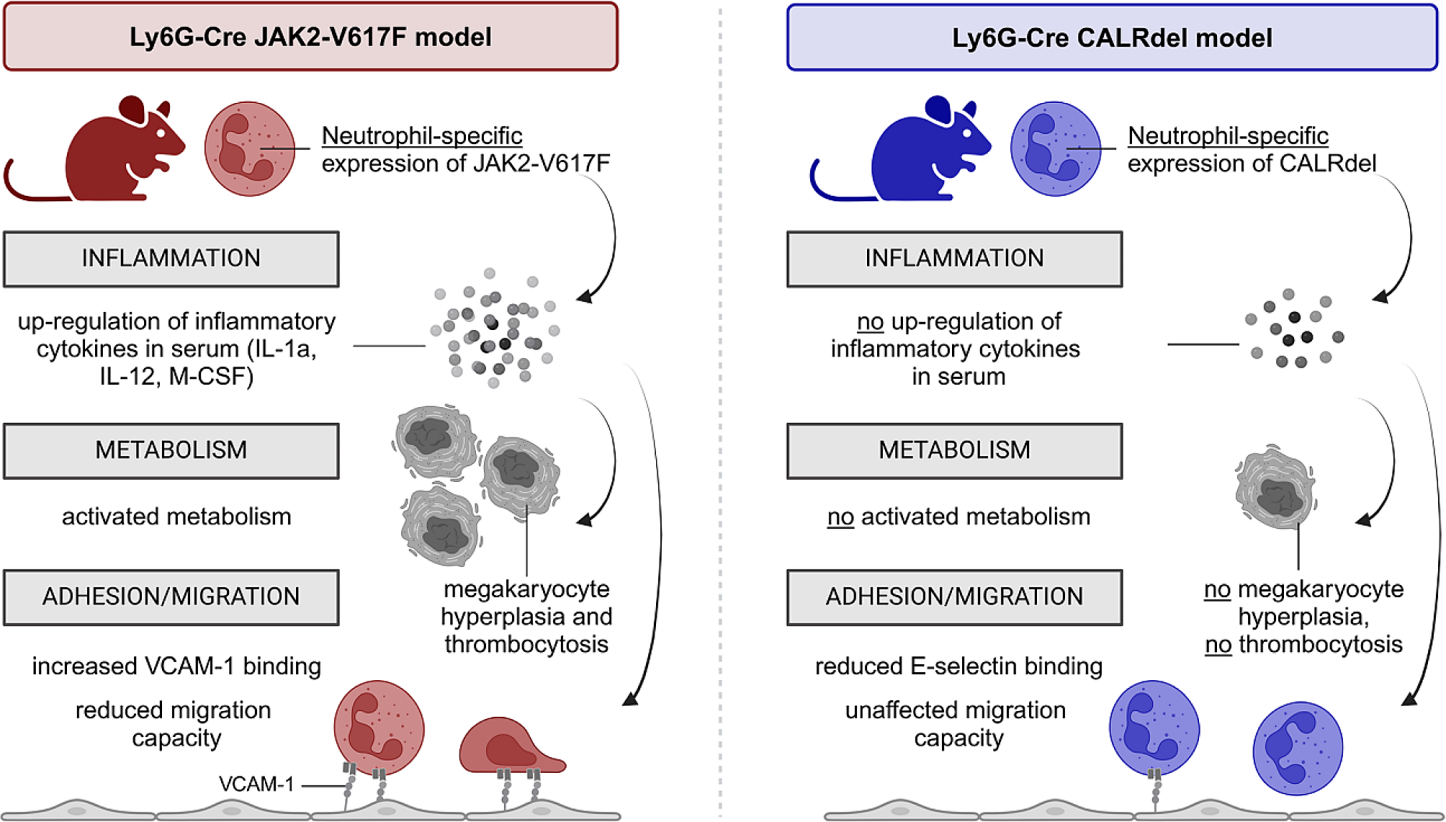
Graphical summary of proposed mechanisms of action of JAK2-V617F in neutrophils. Ly6G-Cre *JAK2*^*+/VF*^ mice exhibit distinct features regarding inflammation, metabolism, adhesion, and migration compared to Ly6G-Cre *CALR*^*+/del*^ mice. Created with Biorender.com

In summary, this study provides a comprehensive analysis of neutrophils carrying JAK2-V617F or CALR mutations. Our findings highlight alterations in inflammatory cytokine production, metabolic functions, and cell migration (summarized in cartoon depicted in Fig. 9) which have implications for the differential pathophysiology of JAK2-V617F versus CALR-mutant disease.

### Electronic supplementary material

Below is the link to the electronic supplementary material.

Supplementary Material 1

Supplementary Material 2

Supplementary Material 3

## Data Availability

The datasets supporting the conclusions of this article are included within the article and its additional files. The associated Source Data can be obtained from the corresponding author upon a reasonable request.

## Abbreviations

MPN: Myeloproliferative neoplasms
ER: Endoplasmic reticulum
SOCE: store-operated calcium entry
TPO: Thrombopoietin
MPL: thrombopoietin receptor
CALRmut: Calreticulin mutation
JAK2: Janus kinase 2
STAT: Signal transducer and activator of transcription
PLTs: Platelets
NEUT: Neutrophil
HCT: Hematocrit
HSPCs: Hematopoietic stem and progenitor cells
MKs: Megakaryocytes
MKPs: Megakaryocyte progenitors
MEP: Megakaryocyte/erythroid progenitors
GSV: Great saphenous vein
TNFα: Tumor Necrosis Factor alpha
GM-CSF: Granulocyte-Macrophage Colony-Stimulating Factor
CXCL: C-X-C Motif Chemokine ligand
CCL: C-C Motif Chemokine ligand
CXCR: C-X-C Chemokine receptor
IL: Interleukin
IL-1ß: Interleukin-1 beta
IL-1α: Interleukin-1 alpha
