# Supplementary material for: Neutrophil-specific expression of JAK2-V617F or CALRmut induces distinct inflammatory profiles in myeloproliferative neoplasia
Source: J Hematol Oncol. 2024 Jun 9;17:43. doi: 10.1186/s13045-024-01562-5 (PMC11163796; doi:10.1186/s13045-024-01562-5)
Supplement: Supplementary file 1 — Supplementary Material 1 [file 13045_2024_1562_MOESM1_ESM.pdf]

Supplemental Tables

Supplemental Table S1A: Overview of primary antibodies used.

Antigen	Conjugate	Clone	Species	Origin	Cat #
B220	Biotin	RA3-6B2	Rat	BioLegend	103203
CD3	Biotin	17A2	Rat	BioLegend	100243
CD4	Biotin	GK1.5	Rat	BioLegend	100403
CD5	Biotin	53-7.3	Rat	BioLegend	100603
CD8	Biotin	53-6.7	Rat	BioLegend	100703
CD9	APC	MZ3	Rat	BioLegend	124811
CD16/32	BV 510	93	Rat	BioLegend	101333
CD18	BV 421	C71/16	Rat	BD Biosciences	562931
CD19	Biotin	6D5	Rat	BioLegend	115503
CD29	APC	HMβ1-1	Armenian Hamster	BioLegend	102215
CD34	FITC	RAM34	Rat	BD Biosciences	553733
CD41	BV 421	MWReg30	Rat	BioLegend	133912
CD42d	APC	IC2	Armenian Hamster	BioLegend	148505
CD49b	Biotin	HMa2	Armenian Hamster	eBioscience	13-0491-85
CD61	APC	2C9.G2	Armenian Hamster	BioLegend	104315
CD150	PE/Cy7	TC15 – 12F12.2	Rat	BioLegend	115913
CD162	BV 421	2PH1	Rat	BD Biosciences	562807
c-Kit	APC/Cy7	2B8	Rat	BioLegend	105825
c-Kit	Biotin	2B8	Rat	BioLegend	105803
c-MPL	Biotin	AMM2	Rat	Immuno-Biological Laboratories	10401
F4/80	Biotin	BM8	Rat	BioLegend	123105
Gr-1	APC	RB6-8C5	Rat	BioLegend	108411
Gr-1	Biotin	RB6-8C5	Rat	BioLegend	108403
IL-7Rα	Biotin	A7R34	Rat	BioLegend	135005
Ly6G	BV 421	1A8	Rat	BioLegend	127627
pSTAT5	Alexa Fluor 647	pY694	Mouse	BD Biosciences	562076
Sca-1	PE/Cy7	D7	Rat	BioLegend	108113
Sca-1	PerCP	D7	Rat	BioLegend	108121
Ter119	Biotin	TER-119	Rat	BioLegend	116203

<u>Supplemental Table S1B</u>: Flow cytometry antibodies to detect intracellular IL-1α in hematopoietic progenitors and mature cell populations

Marker	Clone	Fluorochrome	Dilution	Company	Cat #
CD3	17A2	PE	1:100	BioLegend	100205
CD9	MZ3	PerCP-Cy5.5	1:25	BioLegend	124817
CD11b	M1/70	PerCP	1:100	BioLegend	101229
CD16/32	93	APC-Cy7	1:100	BioLegend	101327
CD16/32	93	BV510	1:100	BioLegend	101333
CD19	6D5	FITC	1:100	BioLegend	115506
CD34	MEC14.7	Pe-Dazzle	1:33	BioLegend	119329
CD41	MWReg30	BV421	1:100	BioLegend	133912
c-kit (CD117)	2B8	PerCP	1:33	BioLegend	105821
SLAM (CD150)	TC15-12F12.2	PE-Cy7	1:100	BioLegend	115913
Ly-6A/E (Sca-1)	D7	Alexa Fluor® 700	1:50	BioLegend	108141
Ly6-C	HK1.4	BV421	1:100	BioLegend	128032
Ly6-G	1AS	BV510	1:100	BioLegend	127633
F4/80	BM8	PE-Cy7	1:100	BioLegend	123113
Lineage cocktail	17A2; RB6-8C5; RA3-6B2; Ter-119; M1/70;	Pacific Blue	1:5	BioLegend	133310
IL-1α	ALF-161	PE	1:100	BioLegend	503203
PE Armenian Hamster IgG Isotype Ctrl Antibody (IL-1α)	HTK888	PE	1:100	BioLegend	400907

<u>Supplemental Table S2:</u> Significantly increased concentrations of IL-1α, IL-12 (p40), and M-CSF were observed in serum of Ly6G-Cre *JAK2*$^{+/VF}$ mice

Median cytokine concentrations in serum of Ly6G-Cre *JAK2*$^{+/VF}$ and *JAK2*$^{+/+}$ mice (n=6), fold change and corresponding p-value. Determination by Eve Technologies, Canada (Mouse Cytokine Array/Chemokine Array 32-Plex, duplicate testing) using two-fold diluted serum samples. Calculation required at least three independent values, otherwise it was listed as *not evaluable*. Data are shown as median values (unpaired, two-tailed *t*-test).

Cytokine	Ly6G-Cre *JAK2*$^{+/VF}$ mice, median [pg/ml]	Ly6G-Cre *JAK2*$^{+/+}$ mice, median [pg/ml]	Fold change	p-value
Eotaxin	935.03	1065,48	0.878	0.3950
G-CSF	636.37	454.83	1.399	0.9737
GM-CSF	not evaluable	not evaluable	-	-
IFNγ	2.06	3.17	0.650	0.3368
IL-1α	180.97	97.98	1.847	0.0317
IL-1β	5.30	2.62	2.021	0.7414
IL-2	19.31	9.04	2.137	0.3511
IL-3	0.28	0.21	1.333	0.5574
IL-4	0.55	1.08	0.509	0.0637
IL-5	27.15	42.32	0.642	0.4884
IL-6	2.92	13.15	0.222	0.0890
IL-7	1.51	1.75	0.863	0.9119
IL-9	13.93	14.99	0.929	0.3410
IL-10	6.40	3.18	2.013	0.6625
IL-12 (p40)	13.67	8.82	1.550	0.0446
IL-12 (p70)	10.23	16.54	0.619	0.3825
IL-13	38.76	40.82	0.950	0.3407
IL-15	51.65	40.42	1.278	0.6872
IL-17	5.22	3.19	1.639	0.3708
IP-10	129.40	165.56	0.782	0.0185
KC	392.16	356.64	1.100	0.3748
LIF	1.07	0.84	1.281	0.3366
LIX	2944.25	2490.84	1.182	0.6635
MCP-1	56.28	72.73	0.774	0.3284
M-CSF	3.74	1.77	2.113	0.0481
MIG	29.71	35.32	0.841	0.4593
MIP-1α	100.22	74.38	1.374	0.3636
MIP-1β	84.81	93.16	0.910	0.3315
MIP-2	165.72	164.03	1.010	0.8871
RANTES	33.02	33.93	0.973	0.9672
TNFα	8.75	4.45	1.966	0.3307
VEGF	0.93	0.84	1.101	0.3411

<u>Supplemental Table S3:</u> No significant alterations of cytokine concentrations were observed in serum of Ly6G-Cre *CALR*$^{+/del}$ mice

Median cytokine concentrations in serum of Ly6G-Cre *CALR*$^{+/del}$ (n=10) and *CALR*$^{+/+}$ mice (n=8), fold change and corresponding p-value. Determination by Eve Technologies, Canada (Mouse Cytokine Array/Chemokine Array 32-Plex, duplicate testing) using two-fold diluted serum samples. Calculation required at least three independent values, otherwise it was listed as *not evaluable*. Data are shown as median values (unpaired, two-tailed *t*-test).

Cytokine	Ly6G-Cre *CALR*$^{+/del}$ mice, median [pg/ml]	Ly6G-Cre *CALR*$^{+/+}$ mice, median [pg/ml]	Fold change	p-value
Eotaxin	1155.93	1022.83	1.13	0.9929
G-CSF	461.67	515.2	0.90	0.1977
GM-CSF	30.40	34.94	0.87	0.4563
IFNγ	3.58	4.16	0.86	0.9257
IL-1α	332.06	328.19	1.01	0.8376
IL-1β	14.63	19.6	0.75	0.0664
IL-2	59.26	60.81	0.97	0.9996
IL-3	3.01	2.85	1.06	0.5910
IL-4	1.98	1.47	1.35	0.7760
IL-5	28.97	19.04	1.52	0.0867
IL-6	5.82	5.11	1.14	0.5702
IL-7	7.58	6.91	1.10	0.4371
IL-9	66.6	76.11	0.88	0.4874
IL-10	10.41	12.13	0.86	0.6206
IL-12 (p40)	15.03	16.15	0.93	0.6552
IL-12 (p70)	98.45	97.19	1.01	0.4240
IL-13	49.84	55.22	0.90	0.6608
IL-15	102.57	88.48	1.16	0.7360
IL-17	2.52	3.37	0.75	0.2941
IP-10	65.32	62.34	1.05	0.6450
KC	271.57	333.70	0.81	0.9695
LIF	2.64	2.25	1.17	0.2997
LIX	1689.66	996.55	1.70	0.3678
MCP-1	93.34	136.58	0.68	0.2513
M-CSF	31.59	32.42	0.97	0.2548
MIG	96.18	120.78	0.80	0.2469
MIP-1α	104.57	107.38	0.97	0.9011
MIP-1β	74.77	95.17	0.79	0.7410
MIP-2	361.23	342.79	1.05	0.9116
RANTES	31.02	31.71	0.98	0.3897
TNFα	15.22	19.86	0.77	0.1033
VEGF	1.44	1.32	1.09	0.5157

<u>Supplemental Table S4</u>: Summary of patient and healthy donor characteristics

Patients and healthy donors were consecutively recruited for this study upon written consent. The protocol was approved by the local ethics committee. Abbreviations: ET, essential thrombocytosis; MF, myelofibrosis; PV, polycythemia vera; n.a., not available.

Subject	Gender	Age [years]	Diagnosis	Mutation	Allelic ratio [%]	Treatment
G2	male	65	Healthy donor	-	-	-
G4	female	53	Healthy donor	-	-	-
G11	female	40	Healthy donor	-	-	-
J3	male	75	PV	*JAK2-V617F*	19.37	Phlebotomy, Hydroxyurea
J4	female	54	PV	*JAK2-V617F*	7.75	Phlebotomy
J5	male	57	PV	*JAK2-V617F*	42.60	Phlebotomy
J9	male	51	PV	*JAK2-V617F*	14.20	Phlebotomy
C1	male	76	MF	*CALRins*	n.a.	Regular Transfusions
C3	male	56	ET	*CALRdel*	n.a.	Hydroxyurea

<u>Supplemental Table S5:</u> GENE Set Enrichment Analysis (GSEA) for IL-1 pathways on the RNAseq data sets collected. Comparison: JAK2-V617F-positive patients versus healthy donors.

Analysis of *JAK2-V617F* positive patients versus healthy donors indicated a trend towards an enrichment in IL-1 signaling pathway, IL-1 structural pathway and IL-1 receptor pathway.

**JAK2-V617F patients versus healthy donors**			
**NES**	**Adjusted p-value**	**FDR q-value**	**Gene Set**
1.30	0.10	0.14	IL-1 signaling pathway
1.13	0.23	0.28	IL-1 structural pathway
1.26	0.05	0.10	IL-1 receptor pathway

<u>Supplemental Table S6:</u> Significantly dysregulated genes in neutrophils of Ly6g-Cre *JAK2*$^{+/VF}$ mice.

Mean counts of significantly dysregulated genes in bone marrow neutrophils isolated from Ly6g-Cre *JAK2*$^{+/VF}$ compared to *JAK2*$^{+/+}$ mice (n=3), log2-fold change and corresponding p-adjusted value. Determination by Genewiz/Azenta, Leipzig (RNA-Seq).

Gene name		Ly6g-Cre *JAK2*$^{+/VF}$, mean [counts]	Ly6g-Cre *JAK2*$^{+/+}$, mean [counts]	log2FoldChange (JAK2-V617F vs. CALR)	Adjusted p-value
1	Igkv2-112	189.30	48.38	1.981644286	1.60E-07
2	Ccl6	13036.07	18868.69	-0.533561893	1.58E-06
3	Ighv10-3	185.63	39.87	2.230591561	1.58E-06
4	Gadd45g	1803.38	2626.21	-0.543015983	2.70E-05
5	Dusp6	7712.66	11186.35	-0.53659898	3.06E-05
6	Saa3	1104.06	605.22	0.868658152	5.99E-05
7	Zc3h12c	1158.73	710.75	0.707540929	0.000440556
8	Taf7	1437.60	2008.89	-0.483657166	0.000522129
9	Ighv5-17	339.36	149.80	1.187066337	0.000818494
10	Btg1	48406.74	61991.50	-0.356895135	0.000904758
11	Icam1	3551.40	2648.87	0.423675396	0.002354814
12	Cxcl2	11031.02	17133.08	-0.635330689	0.003045304
13	Slc25a33	1316.86	1772.75	-0.429749566	0.005364603
14	Cxcl3	114.03	224.76	-0.983934397	0.013854379
15	Fos	9303.14	12512.38	-0.427762762	0.013920178
16	Gadd45a	6659.42	8417.17	-0.338016537	0.013920178
17	Osgin1	8966.08	11826.90	-0.399745652	0.013920178
18	Ighv1-42	47.04	132.00	-1.487766619	0.014609451
19	Pknox1	1380.83	1779.92	-0.366189491	0.015881742
20	Eif5	17399.37	21958.98	-0.33588731	0.015881742
21	Cxcr4	20855.74	25732.66	-0.303197863	0.015881742
22	H2-Q10	3044.22	4325.47	-0.507216707	0.015881742
23	Phf1	2607.41	3407.18	-0.386070826	0.018055645
24	Mbp	4159.01	5168.98	-0.313745684	0.018055645
25	Cebpb	13388.58	16733.54	-0.32184789	0.018055645
26	Ing2	728.04	1002.15	-0.461207735	0.018055645
27	Iars	1371.76	1020.45	0.427177061	0.018673966
28	Phldb1	490.07	296.72	0.727103625	0.018673966
29	Atg2a	8241.80	10696.30	-0.376322278	0.020512817
30	Pde4b	6191.18	7542.55	-0.284978158	0.020512817
31	Tbc1d10c	1447.83	1846.20	-0.350483555	0.020512817
32	Sh3pxd2b	711.87	495.29	0.525669288	0.020512817
33	Ighv14-2	229.59	110.70	1.050619457	0.020512817
34	Ninj1	4150.08	5408.88	-0.382387333	0.024621133
35	Rabgef1	4362.95	5500.42	-0.334562134	0.025407451
36	Dmwd	1284.70	1887.59	-0.555563986	0.025407451
37	Stk17b	24376.43	31056.14	-0.349434119	0.028532965
38	Neurl3	11701.34	14796.40	-0.338702801	0.028644062
39	Cd83	1201.79	887.00	0.438839548	0.029650626
40	Per1	6900.71	9188.23	-0.413128541	0.02977847
41	Flnb	1058.59	762.14	0.47390638	0.032810286
42	Ets2	14248.93	17356.75	-0.284708582	0.034810066

43	Il1rl1	508.27	365.38	0.477446887	0.035504181
44	Csf2rb2	3918.29	2909.44	0.429862674	0.035504181
45	Dusp1	14472.61	19579.64	-0.436130914	0.038926955
46	Rgs2	12473.08	16079.61	-0.366487941	0.038926955
47	Cytip	10198.56	12328.61	-0.273744535	0.040489687
48	Map3k15	1498.18	1931.16	-0.366497639	0.041325397
49	Ighv1-82	287.37	146.03	0.96898271	0.043159054
50	Carns1	650.42	887.59	-0.448854442	0.043415662
51	Preb	7825.89	9689.66	-0.308424154	0.047223983
52	Socs3	4279.93	3525.69	0.279874584	0.047223983
53	Ypel3	4937.85	5984.90	-0.277287828	0.049450744

<u>Supplemental Table S7:</u> A neutrophil-specific *JAK2-V617F* mutation significantly alters the migration behavior of neutrophils in-vivo and in-vitro.

Mean values of accumulated distance, displacement, mean velocity and directness index in in-vitro time-lapse recording of 200 tdTomato$^+$ neutrophils of Ly6G-Cre *JAK2*$^{+/VF}$ and *JAK2*$^{+/+}$ mice (each n=4) and of 150 tdTomato$^+$ neutrophils of Ly6G-Cre *CALR*$^{+/del}$ and *CALR*$^{+/+}$ mice (each n=3) as well as in intravital 2P microscopy of 2,334 tdTomato$^+$ neutrophils of Ly6G-Cre *JAK2*$^{+/VF}$ and 2,339 tdTomato$^+$ neutrophils Ly6G-Cre *JAK2*$^{+/+}$ mice (n=3). Data are shown as mean±SEM (unpaired, two-tailed t-test).

**in-vitro time-lapse recording**	**Ly6G-Cre *JAK2*$^{+/VF}$ mice, mean±SEM**	**Ly6G-Cre *JAK2*$^{+/+}$ mice, mean±SEM**	**p-value**
Accumulated distance [µm]	81.87±1.842	96.18±1,993	<0.0001
Displacement [µm]	9.302±0.5794	13.63±0.7891	<0.0001
Mean velocity [µm/sec]	0.02213±0.0004979	0.026±0.0005387	<0.0001
Directness index	0.1091±0.004982	0.1354±0.006551	0.0015
**in-vitro time-lapse recording**	**Ly6G-Cre *CALR*$^{+/del}$ mice, mean±SEM**	**Ly6G-Cre *CALR*$^{+/+}$ mice, mean±SEM**	**p-value**
Accumulated distance [µm]	58.92±3.74	60.8±3.599	0.7182
Displacement [µm]	14.51±1.293	15±1.41	0.7943
Mean velocity [µm/sec]	0.01657±0.001052	0.0171±0.001012	0.7182
Directness index	0.2525±0.012	0.2337±0.01069	0.2428
**intravital 2P microscopy**	**Ly6G-Cre *JAK2*$^{+/VF}$ mice, mean±SEM**	**Ly6G-Cre *JAK2*$^{+/+}$ mice, mean±SEM**	**p-value**
Accumulated distance [µm]	79.44±0.9432	99.68±1.089	<0.0001
Displacement [µm]	9.992±0.137	10.76±0.157	<0.0001
Mean velocity [µm/sec]	0.3651±0.002874	0.464±0.003675	<0.0001
Directness index	0.1502±0.00227	0.1231±0.001825	<0.0001
Mean sphericity	0.7833±0.00171	0.7707±0.001513	<0.0001
