# Supplementary material for: Neutrophil-specific expression of JAK2-V617F or CALRmut induces distinct inflammatory profiles in myeloproliferative neoplasia
Source: J Hematol Oncol. 2024 Jun 9;17:43. doi: 10.1186/s13045-024-01562-5 (PMC11163796; doi:10.1186/s13045-024-01562-5)
Supplement: Supplementary file 2 — Supplementary Material 2 [file 13045_2024_1562_MOESM2_ESM.pdf]

Supplemental Methods

Genotyping of Ly6G-Cre JAK2-V617F and CALRdel mice

Genotyping of Ly6G-Cre JAK2-V617F mice was performed by PCR using primers 5'-CGTGCATAGTGTCTGTGGAAGTC-3' (J2KI-1) and 5'-CGTGGAGAGTCTGTAAGGCTAA-3' (J2KI-2) with the following settings: 95°C for 10 minutes followed by 40 cycles of 95°C for 30 seconds, 58°C for 45 seconds and 72°C for 45 seconds. The Cre recombinase-mediated activation of JAK2-V617F in tdTomato expressing neutrophils was verified by excision PCR analysis using primers 5'-GACCAGTTGCTCCAGGGTTA-3' (Neo-Exc-RT) and 5'-TCACAAGCATTTGGTTTTGAAT-3' (J2KI-4) with the following settings: 95°C for 10 minutes followed by 35 cycles of 95°C for 30 seconds, 55°C for 1 minute and 72°C for 1 minute. Unless otherwise stated, experiments were conducted on 10 to 12 weeks old Ly6G-Cre $JAK2^{+/VF}$ and $JAK2^{+/+}$ mice of both sexes with an equal contribution.

Cre recombinase-dependent excision of exon 9 in the floxed CALR allele was confirmed with primers 5'-CCTACCTTCTCAGTGCATCAA-3' (forward) and 5'-ATCTGAACCTGCCTGGAAAA-3' (reverse) with the following settings: 95°C for 3 minutes followed by 35 cycles of 95°C for 30 seconds, 56°C for 30 seconds and 72°C for 1 minute. Unless otherwise stated, experiments were conducted on 10 to 12 weeks old Ly6-Cre $CALR^{+/del}$ and $CALR^{+/+}$ mice of both sexes with an equal contribution.

Isolation of platelets

Platelet-rich plasma was obtained from peripheral blood by centrifugation at 200 g for 20 minutes followed by centrifugation of the supernatant at 200 g for 5 minutes. The supernatant was considered to contain platelet-rich plasma. Platelets were isolated from platelet-rich plasma by centrifugation at 1,000 g for 5 minutes and removing the platelet-poor supernatant. The platelet pellet was resuspended with PBS containing 1% FCS and incubated with platelet markers CD41 and CD61 showing an enrichment of about 78.35±4.1% (n=3) in $CD41^+$ $CD61^+$ platelets. In $CD41^+$ $CD61^+$ platelets of Ly6G-Cre $JAK2^{+/VF}$ and $JAK2^{+/+}$ mice, tdTomato was not expressed (**Figure 1J**). Due to the absence of tdTomato expression, isolated platelets of Ly6G-Cre $JAK2^{+/VF}$ mice were considered to be JAK2-V617F negative.

Promegakaryocyte measurement

A previously described gating strategy was adopted for flow cytometry analysis of murine BM promegakaryocytes.[1]

Megakaryocyte differentiation assay

Lineage-negative bone marrow cells isolated by negative selection from Ly6G-Cre *JAK2*$^{+/+}$ and *JAK2*$^{+/VF}$ mice were differentiated into megakaryocytes according to the differentiation protocol established by Barrachina et al.[2] Hematopoietic progenitor cells were cultured in complete RPMI medium supplemented with TPO (0.5 ng/ml or 2 ng/ml) with and without IL-1β (25 ng/ml) for 4 days. On day 4, CD41 and CD42d expression as well as absolute number of immature CD41+ megakaryocytes and CD41+ CD42d+ mature megakaryocytes were determined in a Cytek© Aurora spectral flow cytometer and normalized with a baseline measurement on day 0.

Measurement of MPL expression

Overall, 1x10^{6} ‚untouched' neutrophils isolated from Ly6G-Cre *CALR*$^{+/+}$ and *CALR*$^{+/del}$ mice (each n=3) were pre-treated with mouse FcR Blocking Reagent (Miltenyi Biotec, 10 minutes, 4°C) and subsequently incubated with a primary biotin-conjugated anti-mouse c-MPL/TPOR monoclonal antibody (Immuno-Biological Laboratories, Clone: AMM2, 1:50, 30 minutes, 4°C). Next, cells were washed and incubated with APC/Cy7-Streptavidin (BioLegend, 1:100, 30 minutes, 4°C). Determination of MFI values for c-MPL on tdTomato^{+} cells was conducted on a Cytek Aurora Spectral Flow Cytometer and corrected by subtracting MFIs of FcR blocked tdTomato^{+} cells incubated with APC/Cy7-Streptavidin only. SYTOX Blue was used for dead cell exclusion.

Measurement of intracellular phosphorylated STAT5

Overall, 1x10^{6} ‚untouched' granulocytes isolated from Ly6G-Cre *CALR*$^{+/+}$ and *CALR*$^{+/del}$ mice (each n=3) were either left untreated or stimulated with 10 ng/ml murine TPO (Peprotech), 10 ng/ml recombinant mouse IL-3 (BioLegend) or both in 37°C for 15 minutes. Subsequently, cells were subjected to fixation with pre-warmed BD Phosflow Fix Buffer I (BD Biosciences) at 37°C for 10 minutes followed by permeabilization with pre-cooled BD Phosflow Perm Buffer III (BD Biosciences) at 4°C for 30 minutes. To eliminate non-specific antibody binding to endogenous Fc receptors, fixed granulocytes were pre-treated with FcR Blocking Reagent (Miltenyi Biotec, 5:100, 10 minutes, 4°C) before being stained with Alexa Fluor 647 mouse anti-phospho-Stat5 (BD Biosciences, Clone: pY694, 5:100, 30 minutes, 4°C). Determination of MFI values for pSTAT5 on tdTomato^{+} cells was conducted on a Cytek© Aurora spectral flow cytometer.

RNA Sequencing

RNA Sequencing was performed by GENEWIZ from Azenta Life Sciences (Leipzig, Germany). Unstimulated bone marrow neutrophils isolated by negative selection from Ly6G-Cre *JAK2*$^{+/+}$, *JAK2*$^{+/VF}$, *CALR*$^{+/+}$ and *CALR*$^{+/del}$ mice or ‚untouched' peripheral blood human neutrophils were lysed in RLT buffer prior to RNA isolation using the RNeasy Mini Kit (Qiagen Hilden, Germany).

Concentration and purity of isolated RNA were quantified using Qubit RNA High Sensitivity Kit (Thermo Fischer Scientific). Significant DNA contamination in human samples was eliminated with DNase treatment prior to library preparation. Removal of ribosomal RNA was accomplished with selection of poly(A) positive transcripts. Analysis of RNA sequencing data provided by GENEWIZ was performed using DESeq2.

Intracellular IL-1α staining

Freshly isolated total bone marrow cells ($1x10^6$ cells) of Vav-Cre *JAK2*$^{+/+}$ or Vav-Cre *JAK2*$^{+/VF}$ mice were washed with PBS + 1% FCS. To differentiate the various cell populations, surface markers were stained as described in Supplementary Table S1B for 15 minutes at room temperature. Cells were washed with PBS + 1% FCS. Cells were fixed, permeabilised and intracellularly stained using BD Cytofix/Cytoperm™ Fixation/Permeabilisation Kit (BD) according to the manufacturer's instructions. For intracellular staining, anti-IL-1α-PE (Biolegend) or PE Armenian Hamster IgG isotype Ctrl antibody (Biolegend) was used (1:100). Mean fluorescence intensity (MFI) was measured by flow cytometry (Cytek® Northern Lights™, Cytek; BD FACSCanto II, BD Bioscience). The specific MFI was calculated by subtracting the MFI of the isotype control from the MFI of the anti-IL-1α-PE antibody stained sample.

Metabolic flux analyses

Bioenergetics of neutrophils were recorded on a Seahorse XFe96 extracellular flux analyzer (Agilent Technologies, St. Clara, CA) as described previously[3,4] by performing a glycolysis stress test and a mitochondrial stress test as recommended by the manufacturer. Briefly, neutrophils were washed in the respective assay buffer and seeded at a density of $3x10^5$/well in a CellTAK™-coated (Corning® Inc., Corning, NY) 96-well Seahorse plate (Agilent Technologies). Extracellular acidification rate (ECAR) as a surrogate for glycolysis was recorded over time upon sequential injection of 10 mM glucose (Agilent Technologies), 1 µM oligomycin, and 100 mM 2DG (both Sigma-Aldrich, St. Louis, MO). Oxygen consumption rate (OCR) as a surrogate for mitochondrial respiration was recorded over time upon sequential injection of 1 µM oligomycin, 1,5 µM FCCP, and 30 µM of each Antimycin a and rotenone (all Sigma-Aldrich). Data was analyzed using the Wave Software 2.6.3 (Agilent Technologies) and Graphpad Prism 9 (GraphPad Software Inc.).

***In-vitro* adhesion assays**

Static adhesion and soluble ligand binding of tdTomato-positive neutrophils were investigated as previously described.[5,6] Briefly, static adhesion was analyzed by using murine Fc-free

ICAM-1 or VCAM-1 (Leinco Technologies) precoated plates. Neutrophils were labeled with calcein AM. Isolated tdTomato^{+} neutrophils resuspended in 1xHBSS were seeded, shortly centrifuged und further incubated over 30 minutes at 37°C. Calcein AM fluorescence was measured through three washing steps using a Synergy HT microplate reader (BioTek). Static adhesion was evaluated as fluorescence, calculated as fold change versus control. Soluble ligand binding was performed using murine Fc-tagged ICAM-1 or VCAM-1 (R&D Systems) and Allophycocyanin (APC) AffiniPure F(ab')$_2$ Fragment Goat Anti-Human IgG, Fcγ fragment specific (Jackson ImmunoResearch) as described previously[5,6]. Soluble ligand binding was evaluated as mean specific fluorescence intensity (MSFI), calculated as fold change versus control.

In-vivo **two-photon (2P) microscopy**

In-vivo two-photon (2P) microscopy was performed as previously described.[7,8] Briefly, imaging was conducted using a LSM 700 laser scanning microscope (Zeiss) with a Mai Tai DeepSee laser (Spectra-Physics) tuned at 980 nm. Emitted signals were split by 625 nm and 555 nm longpass filters and further filtered with 675/35 and 590/25 band pass filters, respectively. Within 120 minutes after application of the partial ligation, time lapses focusing the GSV were taken distal and proximal to the ligature. The vertical span of the acquired volume ranged between 20 and 50 µm, the average frame rate was 4.5 seconds. Analysis of neutrophil cell velocity was performed using Imaris 9.3.1 via generation of surfaces and tracking thereof using the following settings: max distance: 7.5 µm, max gap size: 2, track duration: >120 sec. Among these neutrophil tracks, intravascular neutrophils were selected according to their localization and their motion tracks were analyzed quantitatively.

References for Supplemental Methods

1 Kimmerlin Q, Tavian M, Gachet C, Lanza F, Brouard N. Isolation of Mouse Megakaryocyte Progenitors. *J Vis Exp* 2021 May 20;(171). doi: 10.3791/62498.

2 Barrachina MN, Pernes G, Becker IC, Allaeys I, Hirsch TI, Groeneveld DJ, Khan AO, Freire D, Guo K, Carminita E, Morgan PK, Collins TJ, Mellett NA, Wei Z, Almazni I, Italiano JE, Luyendyk J, Meikle PJ, Puder M, Morgan NV, Boilard E, Murphy AJ, Machlus KR. Efficient megakaryopoiesis and platelet production require phospholipid remodeling and PUFA uptake through CD36. *bioRxiv*. 2023 Feb 12:2023.02.12.527706. doi: 10.1101/2023.02.12.527706. Preprint.PMID: 36798332

3 Faas M, Ipseiz N, Ackermann J, et al. IL-33-induced metabolic reprogramming controls the differentiation of alternatively activated macrophages and the resolution of inflammation. *Immunity*. 2021;54(11):2531-2546.e5.

4 Friščić J, Böttcher M, Reinwald C, et al. The complement system drives local inflammatory tissue priming by metabolic reprogramming of synovial fibroblasts. *Immunity*. 2021;54(5):1002-1021.e10.

5 Gupta N, Edelmann B, Schnoeder TM, et al. JAK2-V617F activates β1-integrin-mediated adhesion of granulocytes to vascular cell adhesion molecule 1. *Leukemia*. 2017;31(5):1223-1226.

6 Edelmann B, Gupta N, Schnoeder TM, et al. JAK2-V617F promotes venous thrombosis through β1/β2 integrin activation. *J Clin Invest*. 2018;128(10):4359-4371.

7 Formaglio P, Alabdullah M, Siokis A, et al. Nitric oxide controls proliferation of Leishmania major by inhibiting the recruitment of permissive host cells. *Immunity*. 2021;54(12):2724-2739.e10.

8 Dudeck J, Kotrba J, Immler R, et al. Directional mast cell degranulation of tumor necrosis factor into blood vessels primes neutrophil extravasation. *Immunity*. 2021;54(3):468-483.e5.
