# Supplementary material for: Neutrophil-specific expression of JAK2-V617F or CALRmut induces distinct inflammatory profiles in myeloproliferative neoplasia
Source: J Hematol Oncol. 2024 Jun 9;17:43. doi: 10.1186/s13045-024-01562-5 (PMC11163796; doi:10.1186/s13045-024-01562-5)
Supplement: Supplementary file 3 — Supplementary Material 3 [file 13045_2024_1562_MOESM3_ESM.pdf]

Supplemental Figures

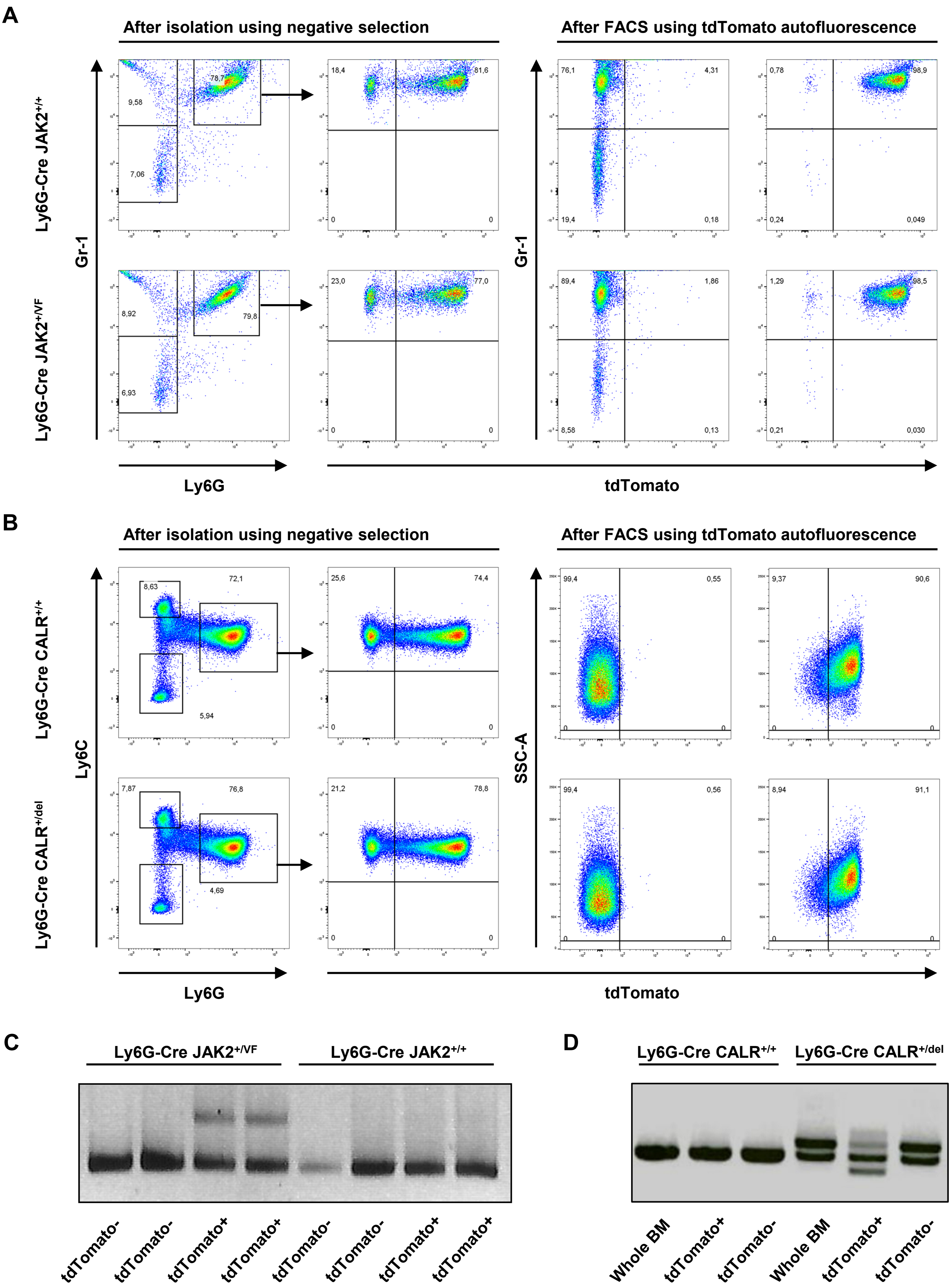
A
After isolation using negative selection
After FACS using tdTomato autofluorescence
Ly6G-Cre JAK2+/+
Ly6G-Cre JAK2+/VF
Gr-1
Ly6G
tdTomato
B
After isolation using negative selection
After FACS using tdTomato autofluorescence
Ly6G-Cre CALR+/+
Ly6G-Cre CALR+/del
Ly6C
SSC-A
Ly6G
tdTomato
C
Ly6G-Cre JAK2+/VF
Ly6G-Cre JAK2+/+
tdTomato-
tdTomato-
tdTomato+
tdTomato+
tdTomato-
tdTomato-
tdTomato+
tdTomato+
D
Ly6G-Cre CALR+/+
Ly6G-Cre CALR+/del
Whole BM
tdTomato+
tdTomato-
Whole BM
tdTomato+
tdTomato-

Figure S1

JAK2-V617F and CALRdel is only expressed in tdTomato-positive neutrophils of Ly6G-Cre *JAK2*$^{+/VF}$ and Ly6G-Cre *CALR*$^{+/del}$ mice, respectively.

(**A**) Left panel: expression of Gr-1, Ly6G, and tdTomato of BM granulocytes isolated from Ly6G-Cre *JAK2*$^{+/+}$ and *JAK2*$^{+/VF}$ mice, respectively upon isolation by negative selection. Right panel: expression of Gr-1 and tdTomato of BM granulocytes isolated from Ly6G-Cre *JAK2*$^{+/+}$ and *JAK2*$^{+/VF}$ mice, respectively after FACS-based cell sorting using tdTomato autofluorescence. FACS sorting using tdTomato autofluorescence resulted in a higher enrichment of tdTomato^{+} cells. (**B**) Left panel: expression of Ly6C, Ly6G, and tdTomato of BM granulocytes isolated from Ly6G-Cre *CALR*$^{+/+}$ and *CALR*$^{+/del}$ mice, respectively upon isolation by negative selection. Right panel: expression of tdTomato in BM granulocytes isolated from Ly6G-Cre *CALR*$^{+/+}$ and *CALR*$^{+/del}$ mice, respectively after FACS sorting using tdTomato autofluorescence. FACS sorting using tdTomato autofluorescence resulted in a higher enrichment of tdTomato^{+} cells. (**C**) Representative excision PCR analysis of isolated tdTomato^{-} and tdTomato^{+} cells of two Ly6G-Cre *JAK2*$^{+/+}$ and *JAK2*$^{+/VF}$ mice. Two distinct PCR bands indicating positivity of JAK2-V617F were found in tdTomato^{+} cells of Ly6G-Cre *JAK2*$^{+/VF}$ mice, only. (**D**) Representative excision PCR analysis of whole BM and isolated tdTomato^{+} and tdTomato^{-} cells of each one Ly6G-Cre *CALR*$^{+/+}$ and *CALR*$^{+/del}$ mouse. Expression of CALRdel is indicated by an additional PCR band in tdTomato^{+} cells of Ly6G-Cre *CALR*$^{+/del}$ mice.

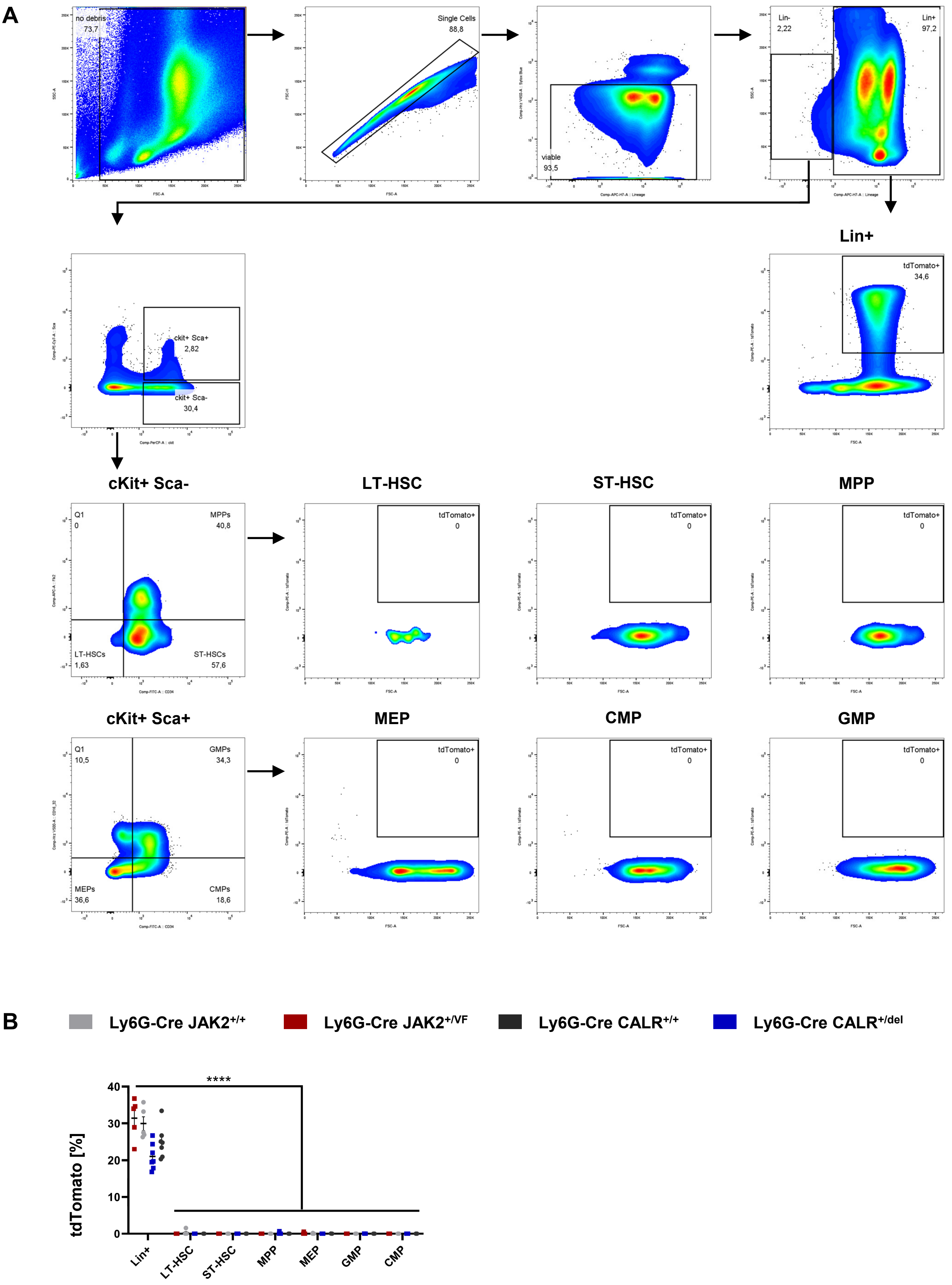
A
no debris
73,7
Single Cells
88,8
viable
93,5
Lin-
2,22
Lin+
97,2
Lin+
tdTomato+
34,6
ckit+ Sca+
2,82
ckit+ Sca-
30,4
cKit+ Sca-
LT-HSC
ST-HSC
MPP
Q1
0
MPPs
40,8
LT-HSCs
1,63
ST-HSCs
57,6
tdTomato+
0
cKit+ Sca+
MEP
CMP
GMP
Q1
10,5
GMPs
34,3
MEPs
36,6
CMPs
18,6
B
Ly6G-Cre JAK2+/+
Ly6G-Cre JAK2+/VF
Ly6G-Cre CALR+/+
Ly6G-Cre CALR+/del
tdTomato [%]
****
Lin+
LT-HSC
ST-HSC
MPP
MEP
GMP
CMP

Figure S2

Hematopoietic stem and progenitor cells (HSPCs) of Ly6G-Cre *JAK2*$^{+/VF}$ and Ly6G-Cre *CALR*$^{+/del}$ mice show negativity for tdTomato, thus negativity for JAK2-V617F and CALRdel, respectively.

(**A**) Representative gating of hematopoietic stem cells (HSC) including long-term (LT-HSC) and short-term hematopoietic stem cells (ST-HSC), multipotent progenitors (MPP), myeloid progenitors (MP), megakaryocyte/erythroid progenitors (MEP), common myeloid progenitors (CMP), and granulocyte/macrophage-progenitors (GMP) of a Ly6G-Cre *JAK2*$^{+/VF}$ mouse. (**B**) All analyzed populations of HSPCs in Ly6G-Cre *JAK2*$^{+/+}$ (n=5), *JAK2*$^{+/VF}$ (n=5), *CALR*$^{+/+}$ (n=7) and *CALR*$^{+/del}$ (n=7) mice show negativity for tdTomato, thus negativity for the expression of JAK2-V617F or CALRdel, respectively. Neutrophils' expression of tdTomato is significantly higher compared to analyzed populations of HSPCs. Data are shown as mean±SEM. ****$p \leq 0.0001$ (unpaired, two-tailed t-test).

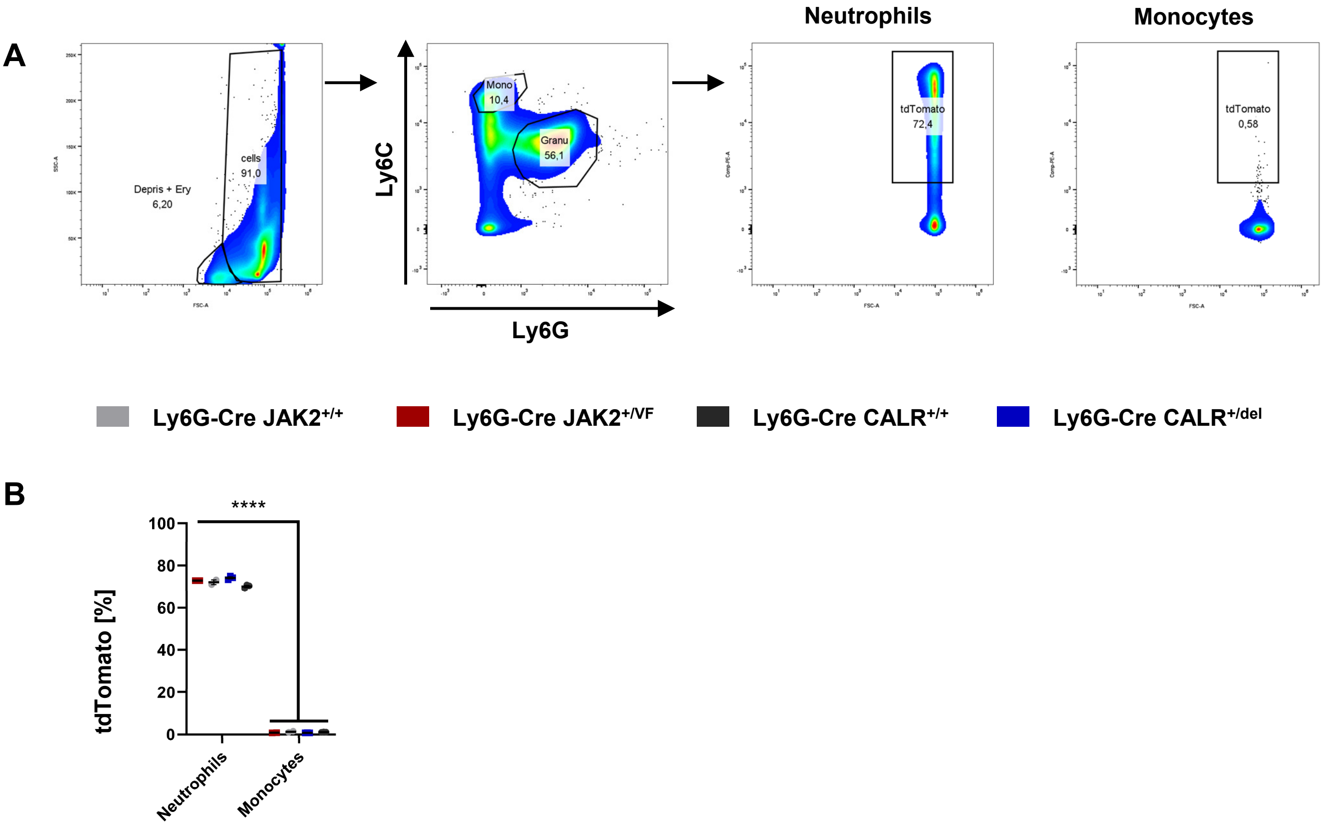
A
Neutrophils
Monocytes
Depris + Ery
6,20
cells
91,0
Ly6C
Ly6G
Mono
10,4
Granu
56,1
tdTomato
72,4
tdTomato
0,58
Ly6G-Cre JAK2+/+
Ly6G-Cre JAK2+/VF
Ly6G-Cre CALR+/+
Ly6G-Cre CALR+/del
B
****
tdTomato [%]
100
80
60
40
20
0
Neutrophils
Monocytes

Figure S3

Monocytes of Ly6G-Cre *JAK2*$^{+/VF}$ and Ly6G-Cre *CALR*$^{+/del}$ mice show negativity for tdTomato, thus negativity for JAK2-V617F and CALRdel, respectively.

(**A**) Representative gating of monocytes and neutrophils using Ly6G and Ly6C of a Ly6G-Cre *JAK2*$^{+/VF}$ mouse. (**B**) Among Ly6G-Cre *JAK2*$^{+/+}$ (n=2), *JAK2*$^{+/VF}$ (n=2), *CALR*$^{+/+}$ (n=3) and *CALR*$^{+/del}$ (n=3), neutrophils' expression of tdTomato is significantly higher compared to monocytes. Monocytes exhibit no expression of tdTomato indicating negativity for JAK2-V617F or CALRdel, respectively. Data are shown as mean±SEM. ****$p \leq 0.0001$ (unpaired, two-tailed t-test).

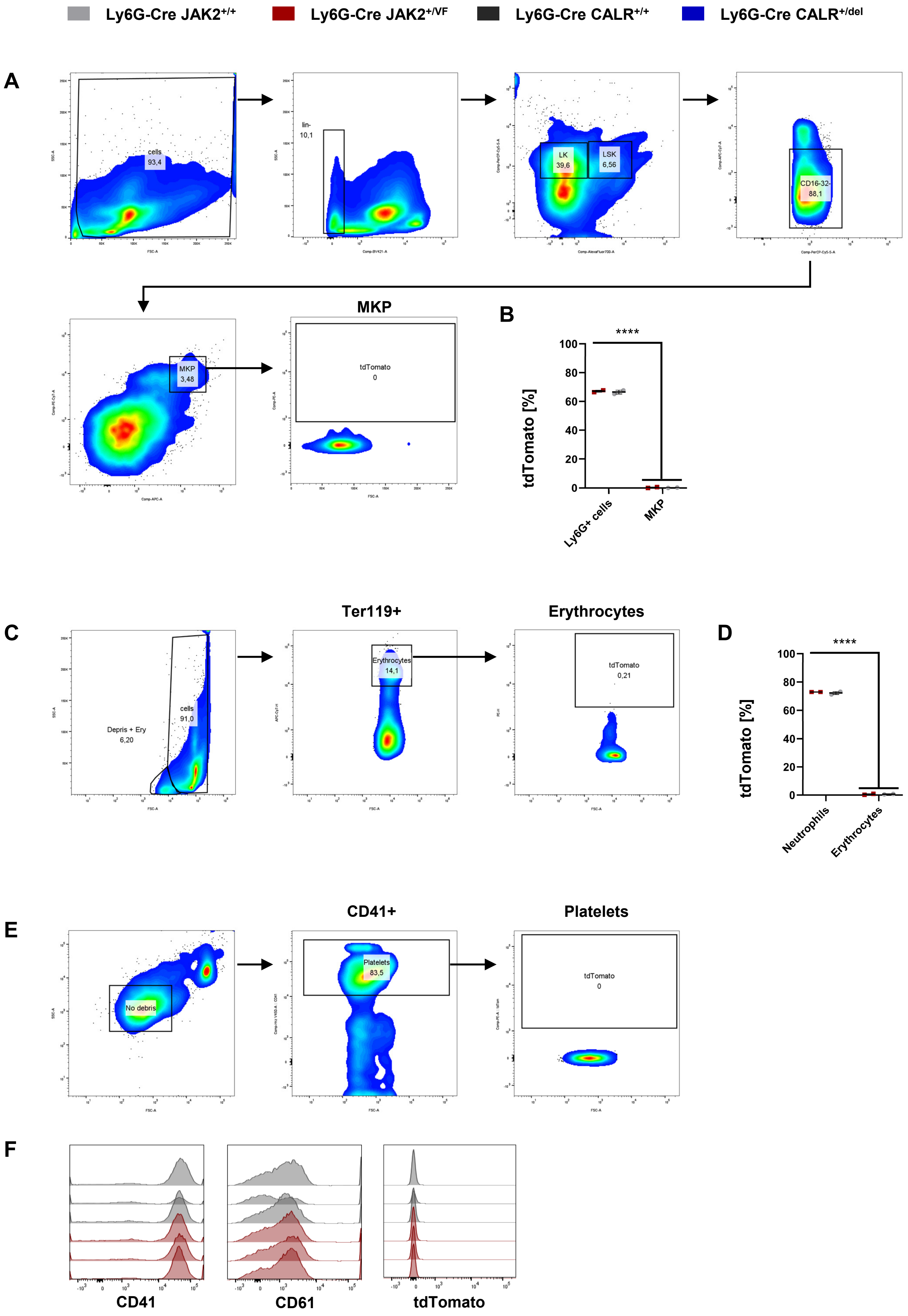
Ly6G-Cre JAK2+/+
Ly6G-Cre JAK2+/VF
Ly6G-Cre CALR+/+
Ly6G-Cre CALR+/del
A
cells
93,4
lin-
10,1
LK
39,6
LSK
6,56
CD16-32-
88,1
MKP
3,48
MKP
tdTomato
0
B
****
tdTomato [%]
Ly6G+ cells
MKP
C
Depris + Ery
6,20
cells
91,0
Ter119+
Erythrocytes
14,1
Erythrocytes
tdTomato
0,21
D
****
tdTomato [%]
Neutrophils
Erythrocytes
E
No debris
CD41+
Platelets
83,5
Platelets
tdTomato
0
F
CD41
CD61
tdTomato

Figure S4

Megakaryocyte progenitors (MKP), erythrocytes and platelets of Ly6G-Cre *JAK2*$^{+/VF}$ mice show negativity for tdTomato, thus negativity for JAK2-V617F´.

(**A**) Representative gating of megakaryocyte progenitors (MKP) of a Ly6G-Cre *JAK2*$^{+/VF}$ mouse. (**B**) Among Ly6G-Cre *JAK2*$^{+/+}$ and *JAK2*$^{+/VF}$ mice (each n=2), expression of tdTomato is significantly higher in Ly6G^{+} cells (neutrophils) compared to MKP. MKP exhibit no expression of tdTomato indicating negativity for JAK2-V617F. Data are shown as mean±SEM. ****p≤0.0001 (unpaired, two-tailed t-test). (**C**) Representative gating of Ter119^{+} erythrocytes of a Ly6G-Cre *JAK2*$^{+/VF}$ mouse. (**D**) Among Ly6G-Cre *JAK2*$^{+/+}$ and *JAK2*$^{+/VF}$ mice (each n=2), expression of tdTomato is significantly higher in neutrophils compared to erythrocytes. Erythrocytes exhibit no expression of tdTomato indicating negativity for JAK2-V617F. Data are shown as mean±SEM. ****p≤0.0001 (unpaired, two-tailed t-test). (**E**) Representative gating of platelets from platelet-rich-plasma of a Ly6G-Cre *JAK2*$^{+/VF}$ mouse. (**F**) Histograms of CD41, CD61, and tdTomato in isolated platelets from peripheral blood of *JAK2*$^{+/+}$ and *JAK2*$^{+/VF}$ mice (n=3). Absent expression of tdTomato indicates JAK2-V617F negativity of isolated platelets of *JAK2*$^{+/VF}$ mice.

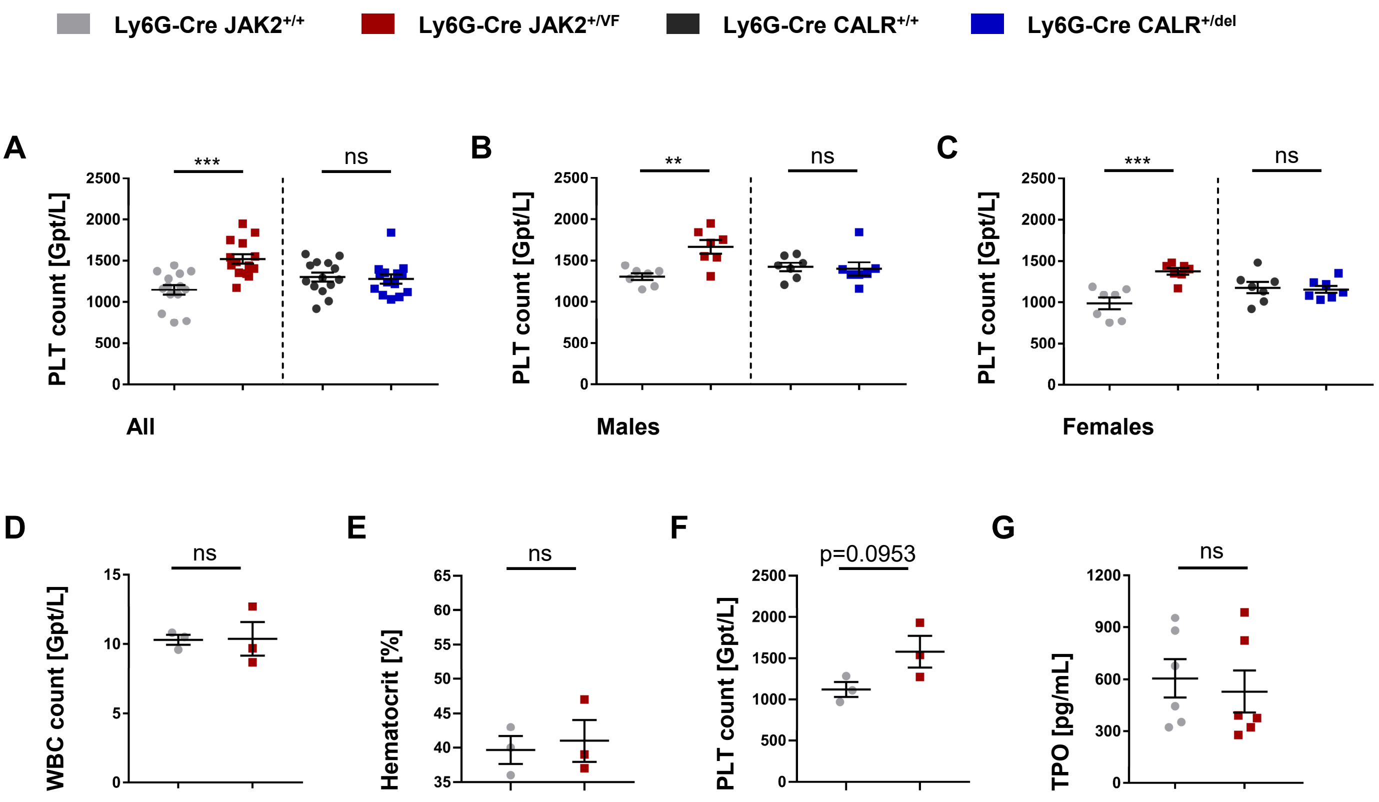

Ly6G-Cre JAK2+/+
Ly6G-Cre JAK2+/VF
Ly6G-Cre CALR+/+
Ly6G-Cre CALR+/del
A
B
C
PLT count [Gpt/L]
***
**
ns
All
Males
Females
D
E
F
G
WBC count [Gpt/L]
Hematocrit [%]
TPO [pg/mL]
p=0.0953

Figure S5

Ly6G-Cre *JAK2$^{+/VF}$* mice show mild but significantly elevated platelet counts regardless of gender.

(**A**) Platelet (PLT) count of Ly6G-Cre *JAK2$^{+/+}$, JAK2$^{+/VF}$, CALR$^{+/+}$* and *CALR$^{+/del}$* mice of both sexes (each n=14) and (**B, C**) separately presented by gender (each n=7). (**D**) White blood cell (WBC) count, (**E**) hematocrit, and (**F**) platelet (PLT) count of aged (older than 30 weeks) Ly6G-Cre *JAK2$^{+/+}$* and *JAK2$^{+/VF}$* mice (each n=3). (**G**) Serum thrombopoietin (TPO) concentrations of Ly6G-Cre *JAK2$^{+/+}$* and *JAK2$^{+/VF}$* mice (each n=6). Data are shown as mean±SEM. **p≤0.01, ***p≤0.001 (unpaired, two-tailed t-test).

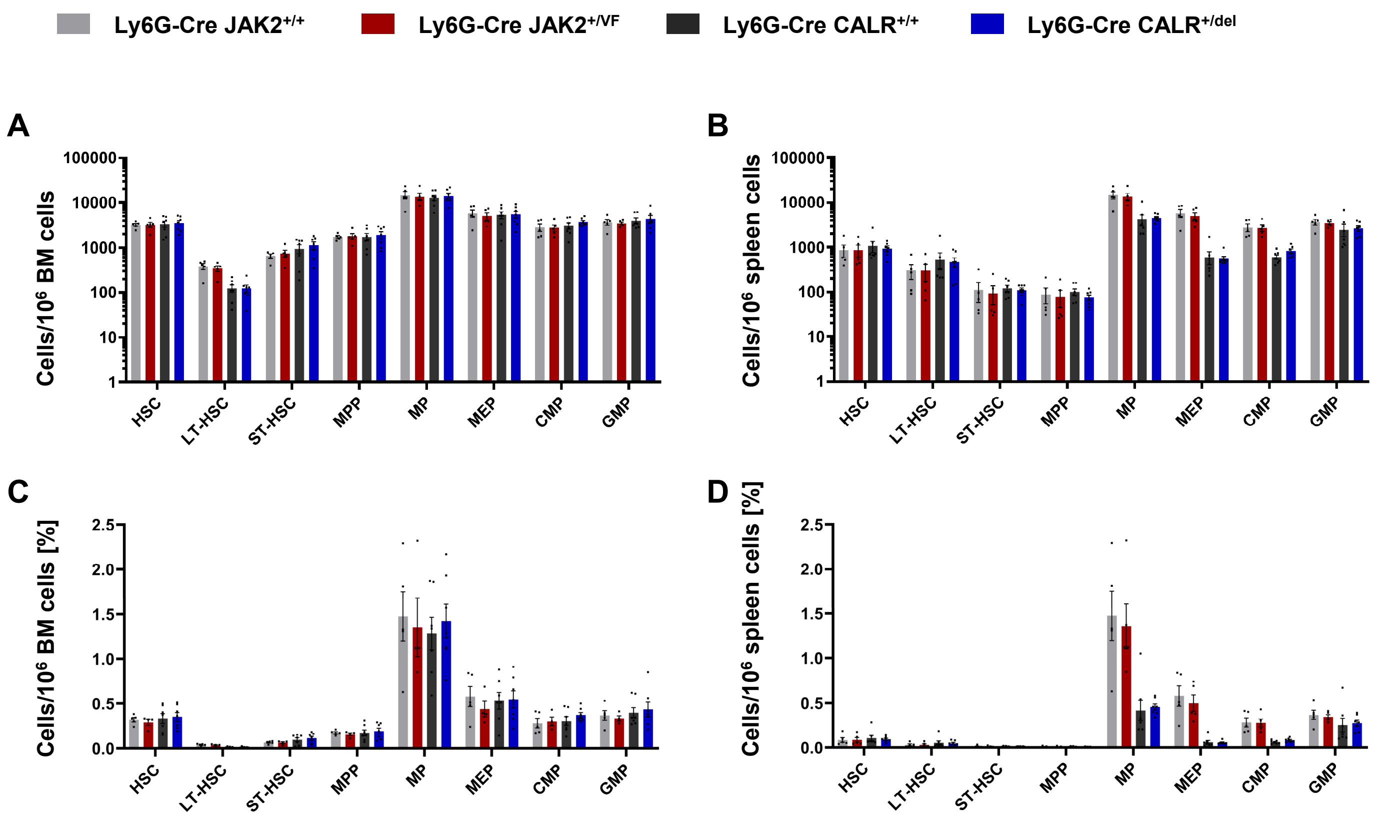

Ly6G-Cre JAK2+/+
Ly6G-Cre JAK2+/VF
Ly6G-Cre CALR+/+
Ly6G-Cre CALR+/del
A
Cells/10⁶ BM cells
B
Cells/10⁶ spleen cells
C
Cells/10⁶ BM cells [%]
D
Cells/10⁶ spleen cells [%]
HSC
LT-HSC
ST-HSC
MPP
MP
MEP
CMP
GMP

Figure S6

Phenotypic analysis of HSPCs reveal that neither JAK2-V617F nor CALRdel expression in neutrophils induce apparent numerical or compositional changes of HSPCs in bone marrow or spleen.

(**A-D**) Phenotypic analysis of hematopoietic stem cells (HSC), long-term (LT-HSC) and short-term hematopoietic stem cells (ST-HSC), multipotent progenitors (MPP), myeloid progenitors (MP), megakaryocyte/erythroid progenitors (MEP), common myeloid progenitors (CMP), and granulocyte/macrophage-progenitors (GMP) in BM and spleen of Ly6G-Cre $JAK2^{+/+}$ (n=5), $JAK2^{+/VF}$ (n=5), $CALR^{+/+}$ (n=7) and $CALR^{+/del}$ mice (n=7). (**A, B**) Absolute and (**C, D**) percentage counts of HSPCs/10^6 BM or spleen cells. Data are shown as mean±SEM.

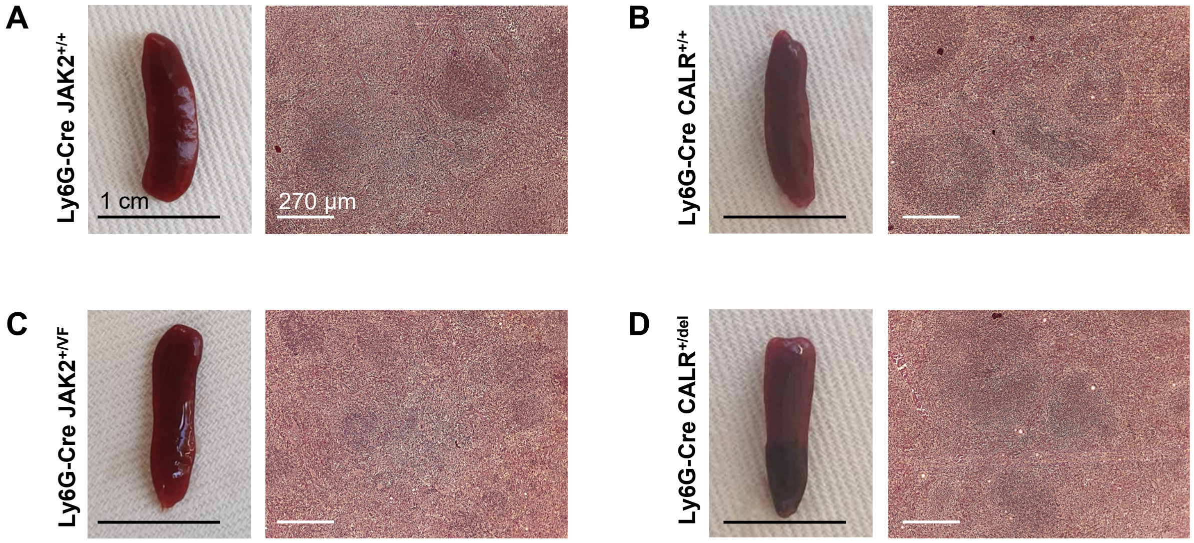
A
Ly6G-Cre JAK2+/+
1 cm
270 µm
B
Ly6G-Cre CALR+/+
C
Ly6G-Cre JAK2+/VF
D
Ly6G-Cre CALR+/del

Figure S7

Spleen size and splenic architecture remain unchanged in Ly6G-Cre *JAK2*$^{+/VF}$ and *CALR*$^{+/del}$ mice.

(**A-D**) Representative photographs of spleens and of hematoxylin-eosin stained spleen sections from Ly6G-Cre *JAK2*$^{+/+}$ (n=5), *CALR*$^{+/+}$ (n=9), *JAK2*$^{+/VF}$ (n=5) and *CALR*$^{+/del}$ mice (n=10).

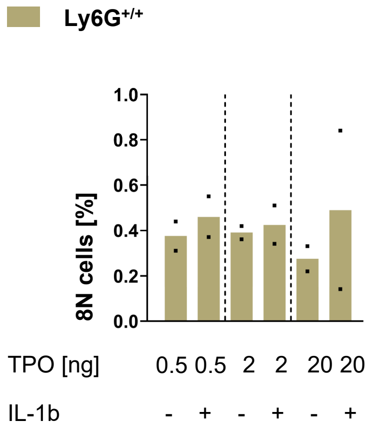
Ly6G+/+
8N cells [%]
1.0
0.8
0.6
0.4
0.2
0.0
TPO [ng]
0.5 0.5 2 2 20 20
IL-1b
- + - + - +

Figure S8

Ploidy analysis indicate an increase in 8N-cells following IL-1β stimulation.

Frequency of N8 megakaryocytes differentiated from lineage-negative cells isolated from BM of *Ly6g*$^{+/+}$ mice (n=2) upon four-day TPO-driven differentiation (0.5, 2 or 20 ng/ml) with or without IL-1β (25 ng/ml). Data are shown as mean.

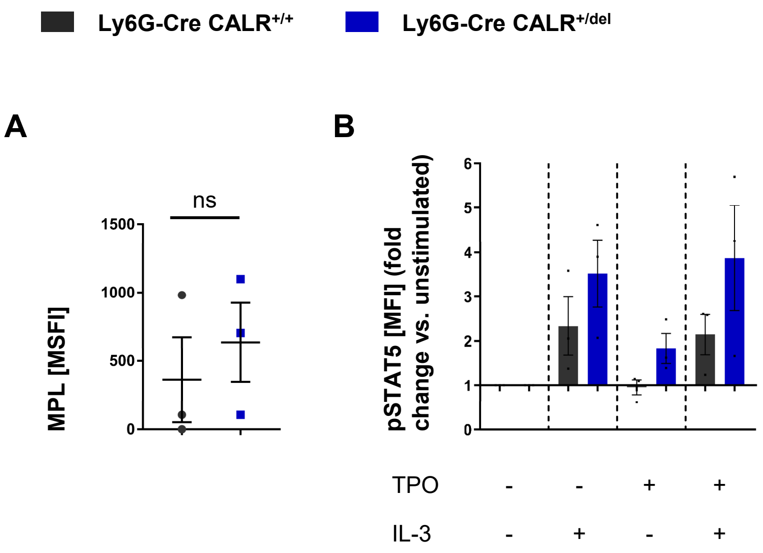
Ly6G-Cre CALR+/+
Ly6G-Cre CALR+/del
A
ns
MPL [MSFI]
1500
1000
500
0
B
pSTAT5 [MFI] (fold change vs. unstimulated)
6
5
4
3
2
1
0
TPO
IL-3
- - + +
- + - +

Figure S9

Analysis of MPL expression and pSTAT5 in BM-derived neutrophils from Ly6G-Cre *CALR*$^{+/+}$ and *CALR*$^{+/del}$ mice.

(**A**) MPL expression on tdTomato^{+} neutrophils isolated from Ly6G-Cre *CALR*$^{+/+}$ and *CALR*$^{+/del}$ mice (each n=3). A negative value indicating absence of MPL expression was converted to zero. (**B**) Phosphorylation of intracellular STAT5 (pY694) in ‚untouched' granulocytes isolated Ly6G-Cre *CALR*$^{+/+}$ and *CALR*$^{+/del}$ mice upon stimulation with murine TPO (10 ng/ml) and/or 10 ng/ml mouse IL-3 (each n=3) expressed as fold change from the unstimulated state. Data are shown as mean±SEM.

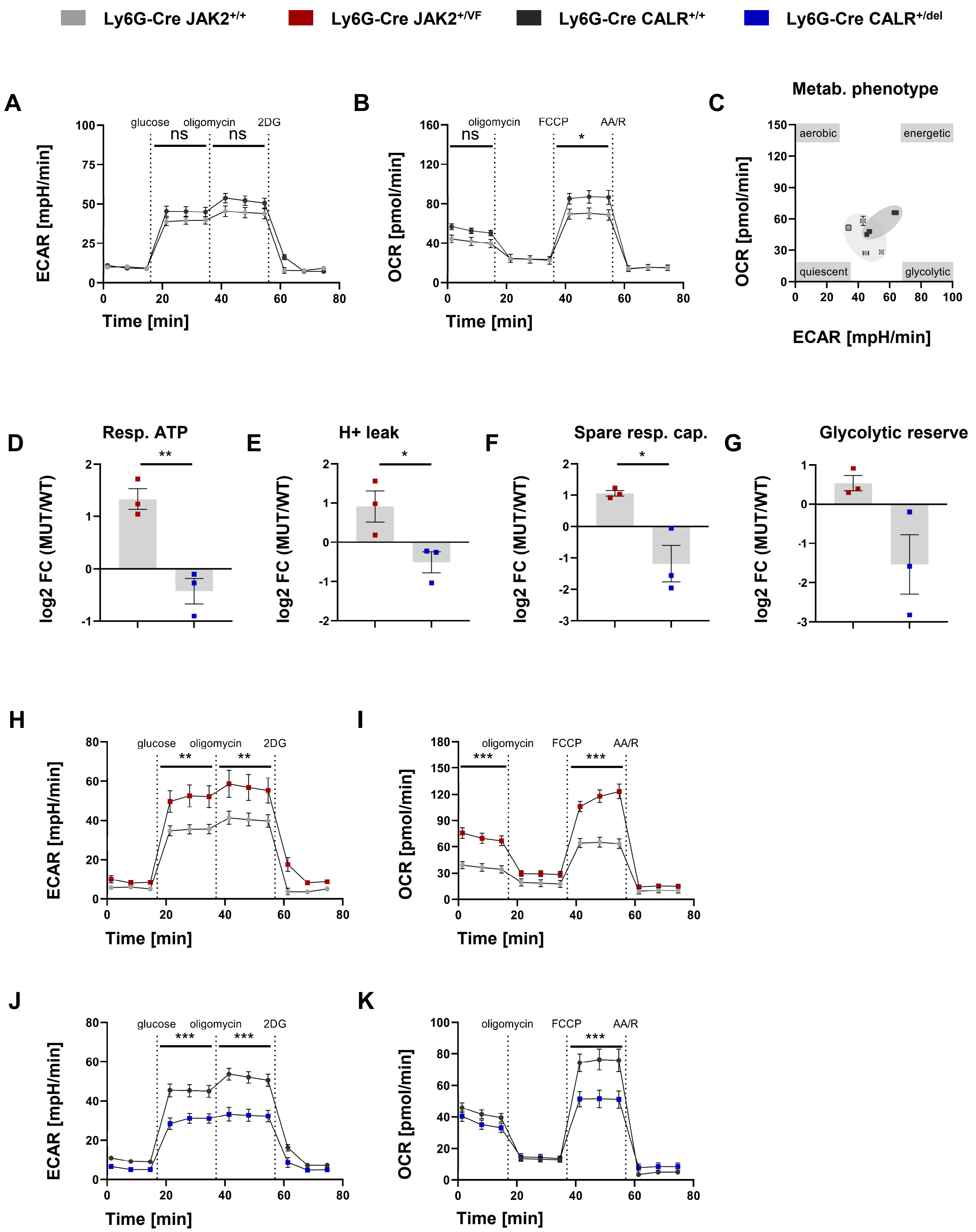

Ly6G-Cre JAK2+/+
Ly6G-Cre JAK2+/VF
Ly6G-Cre CALR+/+
Ly6G-Cre CALR+/del
A
ECAR [mpH/min]
Time [min]
glucose
oligomycin
2DG
ns
ns
B
OCR [pmol/min]
Time [min]
oligomycin
FCCP
AA/R
ns
*
C
Metab. phenotype
OCR [pmol/min]
ECAR [mpH/min]
aerobic
energetic
quiescent
glycolytic
D
Resp. ATP
log2 FC (MUT/WT)
**
E
H+ leak
log2 FC (MUT/WT)
*
F
Spare resp. cap.
log2 FC (MUT/WT)
*
G
Glycolytic reserve
log2 FC (MUT/WT)
H
ECAR [mpH/min]
Time [min]
glucose
oligomycin
2DG
**
**
I
OCR [pmol/min]
Time [min]
oligomycin
FCCP
AA/R
***
***
J
ECAR [mpH/min]
Time [min]
glucose
oligomycin
2DG
***
***
K
OCR [pmol/min]
Time [min]
oligomycin
FCCP
AA/R
***

Figure S10

Additional metabolic parameters of neutrophils isolated from Ly6G-Cre *JAK2*$^{+/+}$ and *JAK2*$^{+/VF}$ mice as well as from Ly6G-Cre *CALR*$^{+/+}$ and *CALR*$^{+/del}$ mice.

(**A**) Glycolysis stress test (GST) and (**B**) mitochondrial stress test (MST) using neutrophils isolated from Ly6g-Cre *JAK2*$^{+/+}$ and Ly6G-Cre *CALR*$^{+/+}$ mice (WT controls) (n=3 with 3-6 technical replicates) corresponding to Fig. 5 were recorded as described and normalized to the background (phase 1 for GST and phase 4 for MST). (**C**) Metabolic phenotype calculated from (**A, B**) and plotted as an ECAR/OCR map corresponding to Fig. 5 H. (**D-G**) Additional metabolic parameters were calculated from data in A, B and Fig. 5 A, B and are presented as the log2 fold change (log2 FC) of each individual relative to the respective WT average. (**H-K**) Glycolysis stress test (GST) (**H, J**) and (**I, K**) mitochondrial stress test using neutrophils isolated from Ly6g-Cre *JAK2*$^{+/VF}$, *JAK2*$^{+/+}$, *CALR*$^{+/del}$ and *CALR*$^{+/+}$ mice. Data are shown as mean±SEM. *p≤0.05, **p≤0.01, ***p≤0.001 (unpaired, two-tailed t-test). Abbreviations: OCR, oxygen consumption rate; ECAR, extracellular acidification rate; 2DG, 2-deoxyglucose; FCCP, Carbonyl cyanide-p-trifluoromethoxyphenylhydrazone; AA/R, Antimycin A/Rotenone.
